# The Phytotoxicity of *Meta*-Tyrosine Is Associated With Altered Phenylalanine Metabolism and Misincorporation of This Non-Proteinogenic Phe-Analog to the Plant's Proteome

**DOI:** 10.3389/fpls.2020.00140

**Published:** 2020-03-06

**Authors:** Hagit Zer, Hila Mizrahi, Nikol Malchenko, Tamar Avin-Wittenberg, Liron Klipcan, Oren Ostersetzer-Biran

**Affiliations:** ^1^Department of Plant and Environmental Sciences, The Alexander Silberman Institute of Life Sciences, The Hebrew University of Jerusalem, Jerusalem, Israel; ^2^Institute of Plant Sciences, the Gilat Research Center, Agricultural Research Organization (ARO), Negev, Israel

**Keywords:** *m*-tyrosine, phenylalanine-tRNA synthetase, mitochondria, chloroplasts, translation, *Arabidopsis thaliana*

## Abstract

Plants produce a myriad of specialized (secondary) metabolites that are highly diverse chemically, and exhibit distinct biological functions. Here, we focus on *meta*-tyrosine (*m*-tyrosine), a non-proteinogenic byproduct that is often formed by a direct oxidation of phenylalanine (Phe). Some plant species (e.g., *Euphorbia myrsinites* and *Festuca rubra*) produce and accumulate high levels of *m*-tyrosine in their root-tips *via* enzymatic pathways. Upon its release to soil, the Phe-analog, *m*-tyrosine, affects early post-germination development (i.e., altered root development, cotyledon or leaf chlorosis, and retarded growth) of nearby plant life. However, the molecular basis of *m*-tyrosine-mediated (phyto)toxicity remains, to date, insufficiently understood and are still awaiting their functional characterization. It is anticipated that upon its uptake, *m*-tyrosine impairs key metabolic processes, or affects essential cellular activities in the plant. Here, we provide evidences that the phytotoxic effects of *m*-tyrosine involve two distinct molecular pathways. These include reduced steady state levels of several amino acids, and in particularly altered biosynthesis of the phenylalanine (Phe), an essential α-amino acid, which is also required for the folding and activities of proteins. In addition, proteomic studies indicate that *m*-tyrosine is misincorporated in place of Phe, mainly into the plant organellar proteomes. These data are supported by analyses of *adt* mutants, which are affected in Phe-metabolism, as well as of *var2* mutants, which lack FtsH2, a major component of the chloroplast FtsH proteolytic machinery, which show higher sensitivity to *m*-tyrosine. Plants treated with *m*-tyrosine show organellar biogenesis defects, reduced respiration and photosynthetic activities and growth and developmental defect phenotypes.

## Introduction

The chemical diversity of terrestrial plants is truly exceptional. Plants are estimated to produce hundreds of thousands of different metabolites, probably the largest number among all other species (see e.g., Moghe and Last, 2015; Wurtzel and Kutchan, 2016; Obata, 2019). Such an exceptional diversity may be a consequence of the large diversification and rapid evolution of specialized metabolic pathways in plants since they occupied the terrestrial environment, about 500 million years ago (Morris et al., 2018). Phytochemicals produced by land-plants are also of great economic and ecological importance, as herbicidal compounds to control weeds.

Phytochemicals, as other natural compounds, are classified into two main categories. Primary metabolites that are associated with essential cellular functions, e.g., nucleotides, amino acids, fatty acids, sugars, and organic acids, which are typically present in all organisms and cells. In addition, organisms also produce numerous specialized (secondary) metabolites that are not essential to basic physiological functions, but otherwise play important roles during specific growth and developmental stages, and aid in adapting and adjusting the developmental need of the plant with specific physiological or environmental signals (Witzany, 2006; Tissier et al., 2014; Moghe and Last, 2015; Wurtzel and Kutchan, 2016; Li et al., 2018). Specialized compounds produced in plants were shown to have key roles in defense mechanisms, in inter- or intracellular signaling, coloring, regulation of primary metabolism, as well as in allelochemisrty (i.e., biomolecules produced by one organism that have a physiological effect on another species when released to the environment) (Rizvi and Rizvi, 1992).

Plants have complex relationships with other organisms, which involve physical and chemical interactions with their surrounding (reviewed by e.g., van Loon, 2016; Bennett and Klironomos, 2018; Mhlongo et al., 2018). These are indicated, for example, by complex interactions between different plants and microorganisms in the soil. Growing roots also need to compete with their neighboring plants, and at the same time to attract beneficial microorganisms to supply them with minerals and nutrients, e.g., root nodules in legumes, where bacterial symbionts fix atmospheric nitrogen (van Loon, 2016; Bennett and Klironomos, 2018; Mhlongo et al., 2018). Accordingly, many chemical signals are exchanged between plants and their neighboring organisms in the soil (Mhlongo et al., 2018). Our study focuses on *meta*-tyrosine (*m*-tyrosine, or m-Tyr), an oxidized byproduct of the aromatic amino acid phenylalanine, which inhibits early post-germination and seedling growth (Bertin et al., 2007; Bertin et al., 2009).

At various conditions and especially under stresses, the production of tyrosine isomers *para-* (i.e., the native amino acid), *meta*-, and *ortho*-tyrosine, can spontaneously occur by the oxidation of the benzyl ring of phenylalanine (Mager and Berends, 1974). A few plant species, including *Euphorbia myrsinites* (donkey-tail spurge) and *Festuca rubra* (red fescue) synthesize enzymatically the isomer *meta*-tyrosine (*m*-tyrosine) in their root tip tissues, and then secrete it to the soil to inhibit nearby plant-life (Mothes et al., 1964; Bertin et al., 2007). In *E. myrsinites*, *m*-tyrosine is produced *via* a transamination of *m*-hydroxyphenylpyruvate (Mothes et al., 1964), whereas the biosynthesis of *m*-tyrosine in *F. rubra* is mediated directly through the hydroxylation of phenylalanine (Bertin et al., 2007; Huang et al., 2012). These data indicate that different plants utilize *m*-tyrosine as a phytotoxic allelochemical using distinct metabolic pathways, activities which likely arose independently during the evolution of land plants (Huang et al., 2012). While *m*-tyrosine is highly toxic to plants, its structural related isomers, *o-* and *p-*tyrosine, have no or only little effects on the germination, growth, or development of land plants (Bertin et al., 2007).

The molecular basis for *m*-tyrosine toxicity has not been resolved yet. We show, by biochemical and genetic approaches, that *m*-tyrosine affects early post-germination development of *Arabidopsis* plantlets by altering the biosynthesis of various amino acids, and in particular the aromatic amino acid Phenylalanine (Phe). Proteomic analyses of young *Arabidopsis* seedlings, grown in the absence or presence of *m*-tyrosine, strongly support that *m*-tyrosine is misincorporated instead of Phe to various organellar (i.e., mitochondrial and plastidial) proteins, an activity which is likely mediated by a dually-localized organellar phenylalanine-transfer RNA (tRNA) synthetase (PheRS) enzyme (Duchêne et al., 2005).

## Materials and Methods

### Plant Material and Growth Conditions

Plants growth and analyses generally followed the procedures described in (Sultan et al., 2016). *Arabidopsis thaliana* ecotype Columbia (Col-0) seeds were obtained from the ABRC center, at Ohio State University (Columbus, OH). *Arabidopsis* mutants of different arogenate dehydratase encoding genes (*ADT1*, At1g11790; *ADT3*, At2g27820; *ADT4*, At3g44720; *ADT5*, At5g22630; *ADT6*, At1g08250) (Chen et al., 2016; Höhner et al., 2018), were generously provided by Prof. Jirong Huang (Shanghai Normal University), while mutants in FtsH2 (At2g30950, also denoted as *var2*) (Takechi et al., 2000; Sakamoto et al., 2003; Zaltsman et al., 2005) were generously given by Prof. Zach Adam (The Hebrew University). We further analyzed the *Arabidopsis atg7-2* (At5g45900; GK-655B06) and *atg5-1* (At5g17290; SAIL-129B079) mutant-lines (Hofius et al., 2009; Yoshimoto et al., 2009; Avin-Wittenberg et al., 2015), which are affected in autophagy. Prior to their germination, the seeds of wild-type and mutant lines were surface sterilized by a vapor-phase method, using a 50 ml sodium hypochlorite (bleach, 6%) solution supplemented with 1.5 ml HCl (37%) solution. The sterilized seeds were sown on Murashige and Skoog (MS)-agar plates, incubated in the dark for 2 days at 4°C, and then transferred to controlled temperature (22°C) and humidity (50%) growth chamber (Percival Scientific, Perry, IA, USA), under short day conditions (8-h light, 250 µE·m-2·s-1 and 16-h dark).

### Microscopic Analyses

For the analysis of plant morphology, plant tissues (i.e., leaves and roots) where obtained from 5-day-old *Arabidopsis* plants grown on MS-plates in the presence or absence of 10 μM *m*-tyrosine. The morphologies of mitochondria and plastids were established by transmission electron microscopy (TEM) of ultrathin plant sections, using Tecnai 12 TEM 100 kV (Phillips, Eindhoven, the Netherlands) microscope equipped with MegaView II CCD camera and Analysis^®^ version 3.0 software (SoftImaging System GmbH, Münstar, Germany), at the Bio-Imaging unit of the Institute of Life Sciences (The Hebrew University of Jerusalem). The relative densities (i.e., pixel intensities) of thylakoid grana membrane stacks and the average surface area of mitochondria have been manually evaluated from TEM images of ultrathin sections of 5-day-old plantlets grown in the absence or presence of *m*-tyrosine, using the ImageJ software (Version 1.52a) (Jensen, 2013). Student's t-test was performed to determine significant differences (P ≤ 0.05).

### Preparation of Crude Organellar Membrane Extracts From *Arabidopsis* Seedlings

Crude organellar proteins were prepared essentially as described previously (Pineau et al., 2008; Shevtsov et al., 2018). In brief, organellar membrane extracts were obtained from 200 mg *Arabidopsis* seedlings (5 days-old), grown in the presence or absence of 10 μM m-tyrosine supplemented to the growth media. The seedlings were then homogenized in 2 ml of 75 mM MOPS-KOH, pH 7.6, 0.6 M sucrose, 4 mM ethylenediaminetetraacetic acid (EDTA), 0.2% polyvinylpyrrolidone-40, 8 mM cysteine, 0.2% bovine serum albumin (BSA), and protease inhibitor cocktail (Roche Diagnostics GmbH, Mannheim, Germany). Protein concentration was determined by the Bradford method (Bio-Rad, Catalog no. 5000201), according to the manufacturer's protocol. For immunoassays, crude membrane fraction were suspended in sample loading buffer (Laemmli, 1970) and subjected to sodium dodecyl sulfate polyacrylamide gel electrophoresis (SDS-PAGE) (at a constant 100 V). Following electrophoresis, the proteins were transferred to a polyvinylidene difluoride (PVDF) membrane (Bio-Rad, Catalog no. 1620177), essentially as described previously (Eubel et al., 2005), and incubated overnight at 4°C with various antibodies (**Table S1**). Detection was carried out by chemiluminescence assay after incubation with an appropriate horseradish peroxidase (HRP)-conjugated secondary antibody.

### Blue Native Gel Electrophoresis for Isolation of Native Organellar Complexes

Blue native (BN)-PAGE of organellar membranous complexes was performed according to the methods described previously (Pineau et al., 2008; Shevtsov et al., 2018). Crude organellar membranes were solubilized with n-dodecyl-ß-maltoside [DDM; 1.5% (w/v)] and loaded onto a native 4 to 16% linear gradient gel. For immunoblotting of non-denaturing PAGE, the proteins were transferred from the gel onto a PVDF membrane (Bio-Rad, Catalog no. 1620177). The membranes were then incubated with specific primary antibodies (**Supplemental Table S1**), and detection was carried out by chemiluminescence assay after incubation with horseradish peroxidase (HRP)-conjugated “secondary” antibodies.

### Proteomic Analyses

Following the extraction of total protein from 5-day-old *Arabidopsis* seedlings and crude organellar preparations (Pineau et al., 2008; Shevtsov et al., 2018), total proteins were obtained by the borate/ammonium acetate method (Maayan et al., 2008). For this purpose, plant tissues were homogenized in the presence of polyvinylpolypyrrolidone (PVPP). The homogenate was added to microfuge tubes containing 400 ml ice-cold protein extraction buffer [50 mM Na-borate, 50 mM ascorbic acid, 1.25% (w/v) sodium dodecyl sulfate (SDS), 12.5 mM β-mercaptoethanol, pH 9.0] and the protease inhibitor cocktail “complete Mini” from Roche Diagnostics GmbH (Mannheim, Germany). Proteins were recovered by centrifugation (25,000 g) in the presence of three volumes of ice-cold 0.1 M ammonium acetate in methanol buffer (NH_4_-OAc-MeOH), following (80% v/v) acetone precipitation. The protein pellet was resuspended with 25 mM Tris-HCl pH 8.0, 10 mM dithiothreitol (DTT), 2% SDS buffer solution. Protein concentration was determined according to the Bradford method, with BSA used as a standard. Twenty-five micrograms of protein was alkylated with 55 mM iodoacetamide (Sigma Chem. Corp. St. Louis, MO) for 30 min at room-temperature in the dark. Removal of SDS followed by digestion with trypsin (Promega Corp., Madison, WS) were performed using the S-Trap microspin column kit (ProtiFi, LLC, Huntington, NY), according to the manufacturer protocol. The tryptic peptides were then desalted, as previously described (Rappsilber et al., 2007). A total of 1.5 µg of peptides from each sample were injected into the mass spectrometer (Q Exactive Plus mass spectrometer, Thermo Fisher Scientific, USA). The data were analyzed using the PEAKS proteomics software, version 8 (Bioinformatics Solutions Inc., Waterloo, ON, Canada), with specified Phe to Tyr replacements (i.e., the mass of *m*-tyrosine is equal to Tyr) (see **Table S3**).

### Amino Acids Analysis

The extraction of *Arabidopsis* plant free amino acids (and various other metabolites) was performed according to a previously described procedure with some modifications by (Roessner-Tunali et al., 2003). In general, 50 mg *Arabidopsis* seedlings (5-day-old) were frozen in liquid nitrogen and homogenized in 700 µl of MeOH, in the presence of 30 µl ribitol (0.2 mg/ml in DDW) as an internal standard. Extraction, derivatization, standard addition, and sample injection by gas chromatography–mass spectrometry (GC-MS) were performed essentially as described previously (Lisec et al., 2006). The GC-MS system comprised of the an Agilent 7693 Autosampler, Agilent J&W DB-35ms column, Agilent 7200B gas chromatograph, quadrupole time-of-flight MS with removable electron ionization source. Amino acids were identified in comparison to commercial standard compounds purchased from Sigma. Chromatograms and mass spectra were evaluated using MassHunter Data Analysis by Agilent, Quantitative and Qualitative Analysis (TOF). Calculations of the relative free amino acid levels were evaluated by calculating the relative pick area of each amino acid in untreated *versus m*-tyrosine grown seedlings. For detailed parameters see **Supplemental Dataset 1** (https://figshare.com/articles/Frontiers_in_Plant_Sciences_MS_data/11627211).

## Results

### *m*-Tyrosine Affects *Arabidopsis* Early Seedlings Establishment and Root Development

Specialized phytochemical compounds are highly diverged chemically, and are expected to arise in plants to aid with key biological functions, such as in surviving and communicating with other organisms in complex environmental niches. *F. rubra*, a grass species that is widespread across the northern hemisphere, releases to the soil biochemicals that inhibit the growth of nearby plant-life. NMR-spectroscopy led to the identification of *m*-tyrosine as the key phytotoxic compound within root exudates of *F. rubra* plants (Bertin et al., 2007). Plants treated with exogenous *m*-tyrosine are characterized by shortened roots and reduced biomass phenotypes [Bertin et al. (2007) and **Supplemental Figure S1**]. The phytotoxic effects of *m*-tyrosine seem to be mediated by mechanisms that are not related to auxin (Bertin et al., 2007). Our analyses further indicate that *Arabidopsis* seedlings germinated in the presence of *m*-tyrosine (i.e., 0 to 320 μM), show post-germination developmental defects, and strong effects on root growth (calculated IC_50_ value of 2.365 μM; i.e., concentrations required to achieve 50% reduction of *Arabidopsis* root growth) (**Supplemental Figure S1** and **Table 1**).

**Table 1:** Effects of *m*-tyrosine on root length and leaf number of 5-day-old *Arabidopsis thaliana* (col-0) seedlings.

*m*-Tyrosine (μM)	Root-length (cm)	No. of leaves^*^1^^
0	2.6 ± 0.19	4 ± 2
2.5	1.19 ± 0.11	4 ± 0
5	0.91 ± 0.15	4 ± 0
10	0.54 ± 0.05	4 ± 0
20	0.10 ± 0.016	2 ± 2
40	0.12 ± 0.017	2 ± 2
80	0.05 ± 0.007	2 ± 0
160	0.05 ± 0.006	2 ± 0
360	0.02 ± 0.003	0 ± 0

The data shows the relative root lengths, number of leaves, total chlorophyll, total protein, respiration, and photosynthetic activities in untreated versus m-tyrosine-treated seedlings. The values are means of 4–7 biological replicates ± SE.

*1—number of leaves including cotyledons.

### Free Amino Acid Analysis of *Arabidopsis* Seedlings Grown on *m*-Tyrosine

**Figure 1:**
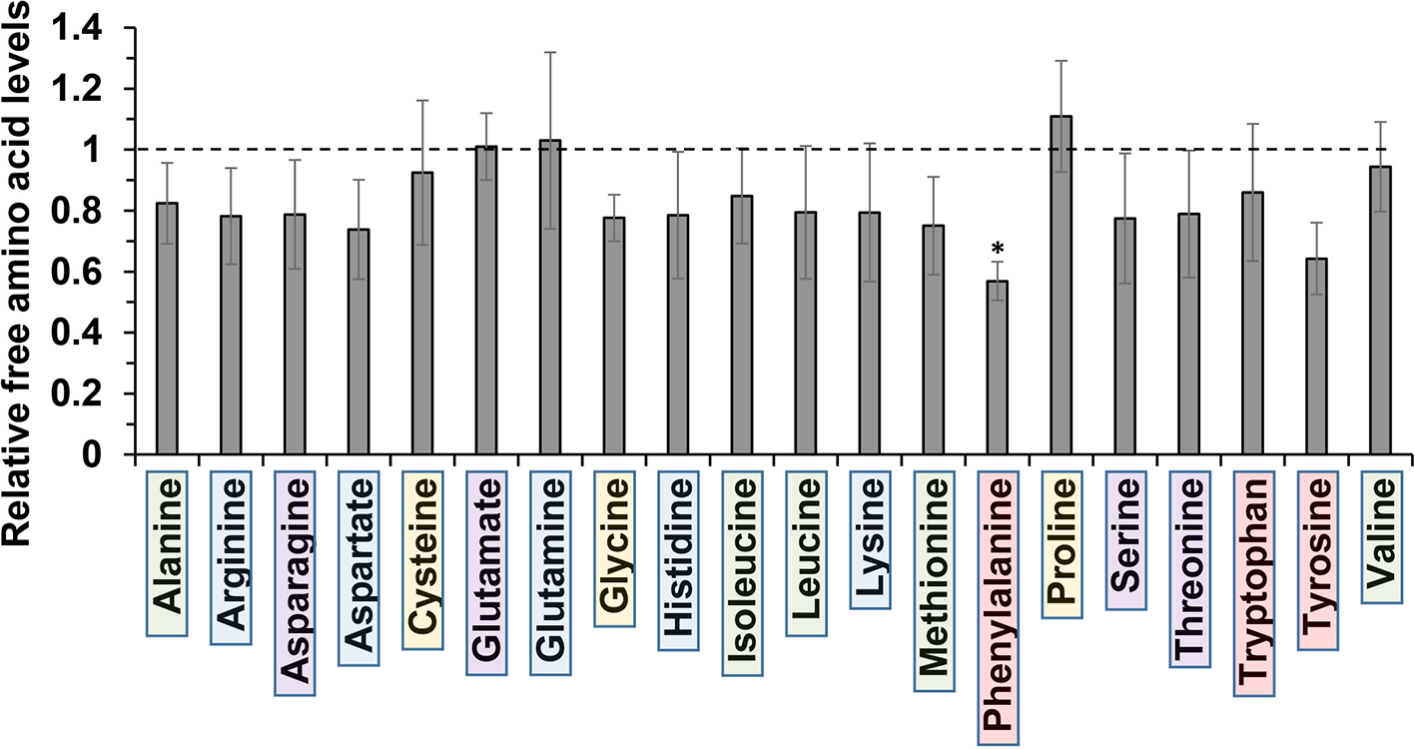
Relative change in free amino acid levels in 5-day-old *Arabidopsis thaliana* seedlings grown in the presence or absence of 10 μM *m*-tyrosine. *Arabidopsis* seeds were germinated in Murashige and Skoog (MS)-agar plates in the absence or presence of 10 μM *m*-tyrosine (*m*-Tyr). Amino acids, extracted from 50 mg of 5-day-old *Arabidopsis* seedlings, were identified in comparison to commercial standard compounds from each treatment (i.e., control and *m*-Tyr-grown seedlings), in the presence of ribitol (0.2 mg/ml) as an internal standard. Extraction, derivatization, standard addition, and sample injection were performed essentially as described previously (Lisec et al., 2006). Calculations of the relative free amino acid levels were evaluated from the relative pick area of each amino acid in untreated *versus m*-tyrosine-treated seedlings. The values are means of four biological replicates, while error bars indicate one standard deviation (see **Table S2**). Color boxes indicate to functional groups: in green hydrophobic side chains, blue for electrically charged side chains, purple for polar (uncharged) side chains, red for aromatic residues, and yellow indicates special side chains.

The molecular basis for *m*-tyrosine mediated phytotoxicity is largely unclear in plants. Given its chemical properties, we speculate that *m*-tyrosine might be incorporated to the plant proteome and/or interferes with cellular metabolism. To address this, we analyzed the relative accumulation of free amino acids in 5-day-old *Arabidopsis thaliana* seedlings grown in the absence (control, −*m*-Tyr) or presence of 10 μM *m*-tyrosine (+*m*-Tyr) by GC-MS analysis (see **Figure 1**, and **Supplemental Table S2** and **S3**). The accumulation of various amino acids was evaluated relatively to the control (non-treated) plants grown under the same growth conditions (see **Table S3**).

Under “normal” growth conditions (see *Materials and Methods*), the steady-state levels of various amino acids, including the nonpolar (hydrophobic) Ala, Ile, Leu, Met, and Val, the uncharged Ser and Thr, positively charged Arg, His, and Lys, the negatively charged Asp, as well as the aromatic residues Trp and Tyr, were all reduced to some extent (i.e., from 7 to 26%) in the *m*-tyrosine germinated seedlings (**Figure 1**, and **Table S2**). However, only Phe showed a statistically significant reduction (43.1 ± 6.3%) in the treated plants (see **Figure 1**, and **Table S2**). The nonpolar amino acid Pro, which is known to accumulate under different stress conditions (Szabados and Savouré, 2010), was found to be somewhat higher (110.9 ± 18.2%) in plants germinated in the presence of *m*-tyrosine, whereas the steady-state levels of the negative charged Glu (1.01 ± 0.14%) and the uncharged Gln (103.0 ± 2.9%) amino acids were not significantly affected by the addition of *m*-tyrosine to the growth media (**Figure 1**, **Table S2**).

**Figure 2:**
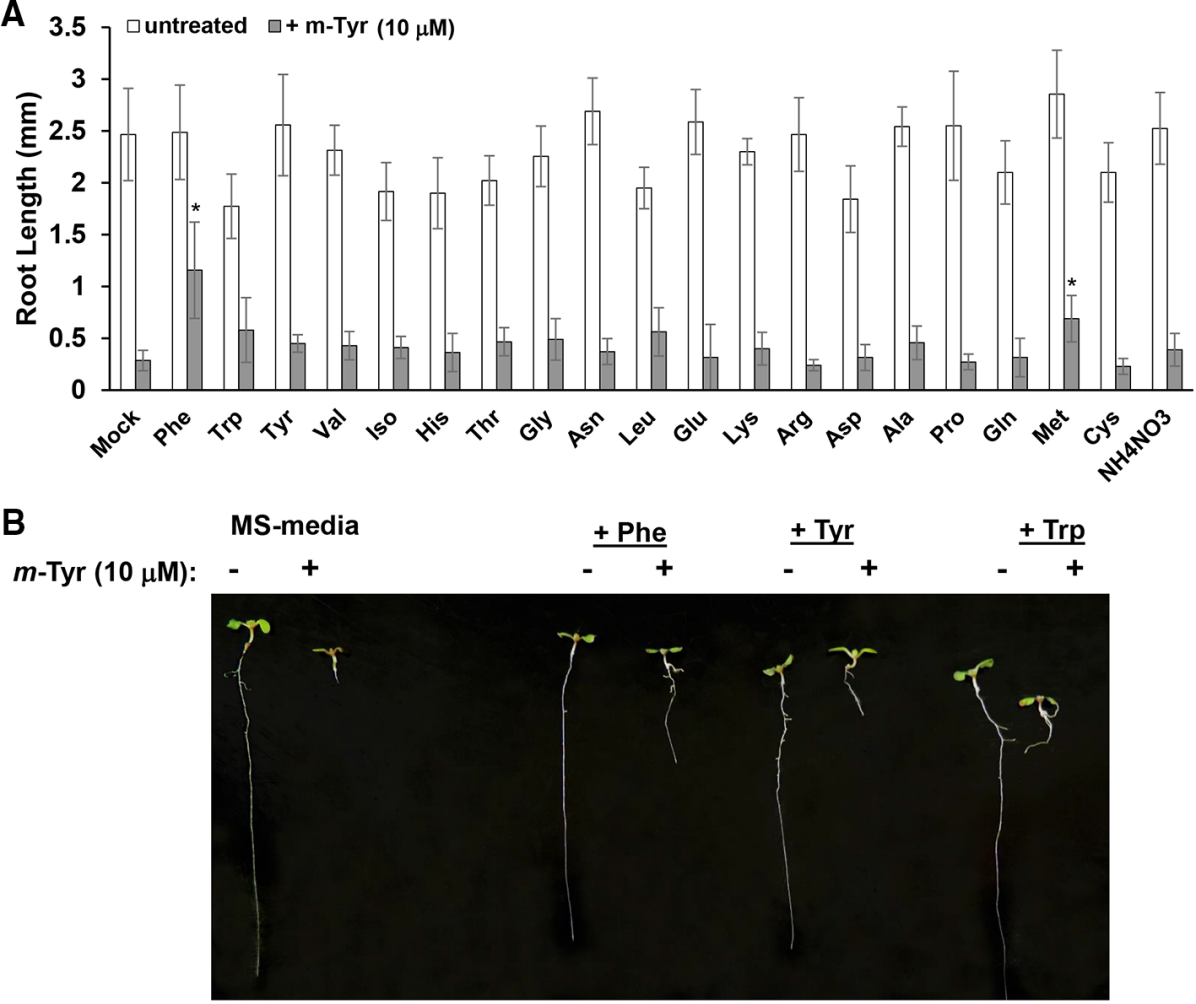
The effect of exogenous amino acids on *m*-tyrosine mediated toxicity. **(A)** Five-day-old *Arabidopsis* seeds were germinated in Murashige and Skoog (MS)-agar plates containing various amino acids (40 μM), in the absence or presence of 10 μM *m*-tyrosine. **(B)** Measurements of the root lengths in 5-day-old *Arabidopsis* seedlings. Bar in (panel **A**) represents 1.0 cm. The values are means of three biological replicates with about 25 seedlings in each treatment. Error bars indicate one standard deviation. Asterisks in (panel **B**) indicate a significant difference from 10 μM *m*-Tyr grown plants (Student's T-test, P ≤ 0.05).

Growing plants in the presence of exogenous amino acids was previously shown to partially restore the growth and developmental defect phenotypes of *Arabidopsis* seedlings treated with *m*-tyrosine (Bertin et al., 2007). Root growth inhibition by 3 μM *m*-tyrosine was counteracted by 14 out of 21 different amino acids given at 40 μM concentrations (Bertin et al., 2007). However, when the amino acids were administered to the growth media together with a higher concentration (i.e., 10 μM) of *m*-tyrosine, only Phe showed a notable “rescue” effect on *Arabidopsis* root growth inhibition (**Figure 2**).

### The Effects of *m*-Tyrosine on Mutants Affected in Arogenate Dehydratase Activity

**Figure 3:**
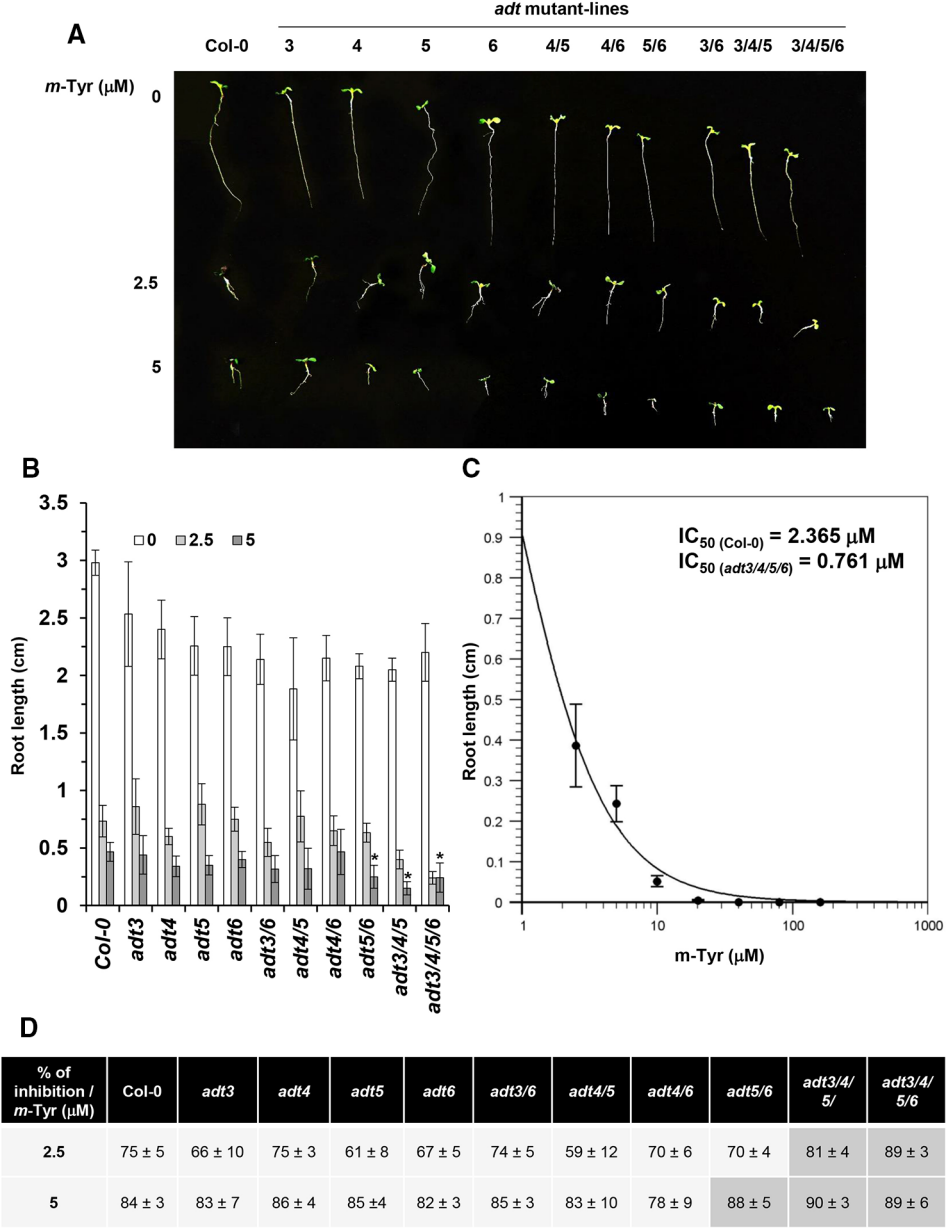
The effect of *m*-tyrosine on *Arabidopsis* wild-type and single, double, triple, or quadruple *adt* knockout mutant-lines. **(A)** Seeds of *Arabidopsis* wild-type (Col-0) and homozygous *adt* mutant-lines (Chen et al., 2016; Höhner et al., 2018) (generously provided by Prof. Jirong Huang, Shanghai Normal University) were germinated in Murashige and Skoog (MS)-agar plates in the absence or presence of different concentration of *m*-tyrosine (i.e., 0, 2.5, and 5 μM), as indicated in the panel. The figure show 5-day-old seedlings. **(B)** Measurements of the root lengths in 5-day-old *Arabidopsis* wild-type (Col-0) plants and *adt* mutants. **(C)** IC_50_ of the quadruple *adt3/4/5/6* mutant was calculated using the ‘Quest-Graph™ IC50 calculator' (https://www.aatbio.com/tools/ic50-calculator). **(D)** The effect of *m*-tyrosine (i.e., % of inhibition) on the relative root length ratios in 5-day-old wild-type (Col-0) plants and *adt* mutants. Bar in (panel **A**) represents 1.0 cm. The values are means of three biological replicates with about 25 seedlings in each treatment. Error bars indicate one standard deviation. Asterisk in (panel **B**) indicates a significant difference from 5 μM *m*-Tyr grown plants (Student's T-test, P ≤ 0.05).

The biosynthesis of Phe in various organisms involves a series of enzymatic reactions, which convert prephanate (a product of the shikimate pathway) into Phe, from either phenylpyruvate or arogenate (the later serves as the primarily route for Phe synthesis in angiosperms) (Cho et al., 2007; Tzin and Galili, 2010). In *Arabidopsis*, arogenate dehydratase (ADT) catalyzes the final step in Phe biosynthesis (i.e., decarboxylation of arogenate), a reaction that takes place within the plastids (Tohge et al., 2013). *Arabidopsis* contains six closely related *ADT* genes, which differ in their organ locations and contribution to Phe accumulation (Höhner et al., 2018). *Arabidopsis* ADT-deficient mutants show altered photosynthetic rates, where stronger phenotypes are seen in lines that had deficiencies in multiple ADT isoforms (Höhner et al., 2018). These data are in accordance with the genetic and biochemical analyses, as well as with the expression patterns of different *ADT* genes in *Arabidopsis*, which indicate that different ADT isoforms in land plants are redundant in Phe-biosynthesis (Chen et al., 2016; Höhner et al., 2018). *Arabidopsis* mutants that accumulate lower levels of free-Phe are expected to show higher sensitivity to *m*-tyrosine toxicity, due to altered Phe metabolism (**Figure 1** and **Table S2**) and/or increased misincorporation of the Phe-analog into the plant proteome. Analysis of single, double, triple, and quadruple knockout lines (Chen et al., 2016; Höhner et al., 2018) (generously provided by Prof. Jirong Huang, Shanghai Normal University) indicated that triple *adt3/4/5* and quadruple *adt3/4/5/6* mutants are more sensitive to *m*-tyrosine, further supporting a functional redundancy between different ADT paralogs in *Arabidopsis thaliana* plants (**Figure 3** and **S2**). In the absence of *m*-tyrosine, the root sizes were similar in 5-day-old wild-type plants and *adt* mutant-lines (i.e., about 3 cm in length; **Figures 3A, B**). Yet, when the seeds were germinated in the presence of 2.5 or 5 μM *m*-tyrosine, root development was more strongly affected in the triple *adt3/4/5* (81% inhibition at 2.5 μM and 89% at 5 μM *m*-Tyr) or quadruple *adt3/4/5/6* mutants (90% inhibition at 2.5 μM and 89% at 5 μM *m*-Tyr) than in wild-type plants or single *adt* mutant lines (i.e., 61~75% inhibition at 2.5 μM and 82~84% at 5 μM *m*-Tyr) (**Figures 3B, D**). Accordingly, the *m*-tyrosine concentration required to achieve 50% reduction of root growth of *adt3/4/5/6* mutant-line was notably lower than that of wild-type plants (i.e., IC_50_ values 0.761 and 2.365 μM, respectively) (**Figures 3C** and **S1**).

Next, we examined the effects of different Phe concentrations (i.e., 20, 40, and 80 μM) on root growth of *Arabidopsis thaliana* wild-type (Col-0) and *adt3/4/5/6* mutants grown in the absence or presence of 10 μM *m*-tyrosine (**Figure S2A**). Increased concentrations of Phe partially rescued the early seedlings establishment and root developmental defect phenotypes by *m*-tyrosine (**Figure S2A**). At concentrations above 40 μM phenylalanine, the *m*-tyrosine mediated altered post-germination development and inhibited root development were restored in both the wild-type and *adt3/4/5/6* mutant (**Figure S2A**). To a lesser degree, the effect of *m*-tyrosine on root growth was also counteracted by the addition of Phe in other *adt* mutant-lines (**Figure S2B**).

### *Arabidopsis* Plants Treated With *m*-Tyrosine Display Chloroplasts and Mitochondria Biogenesis Defect Phenotypes

**Figure 4:**
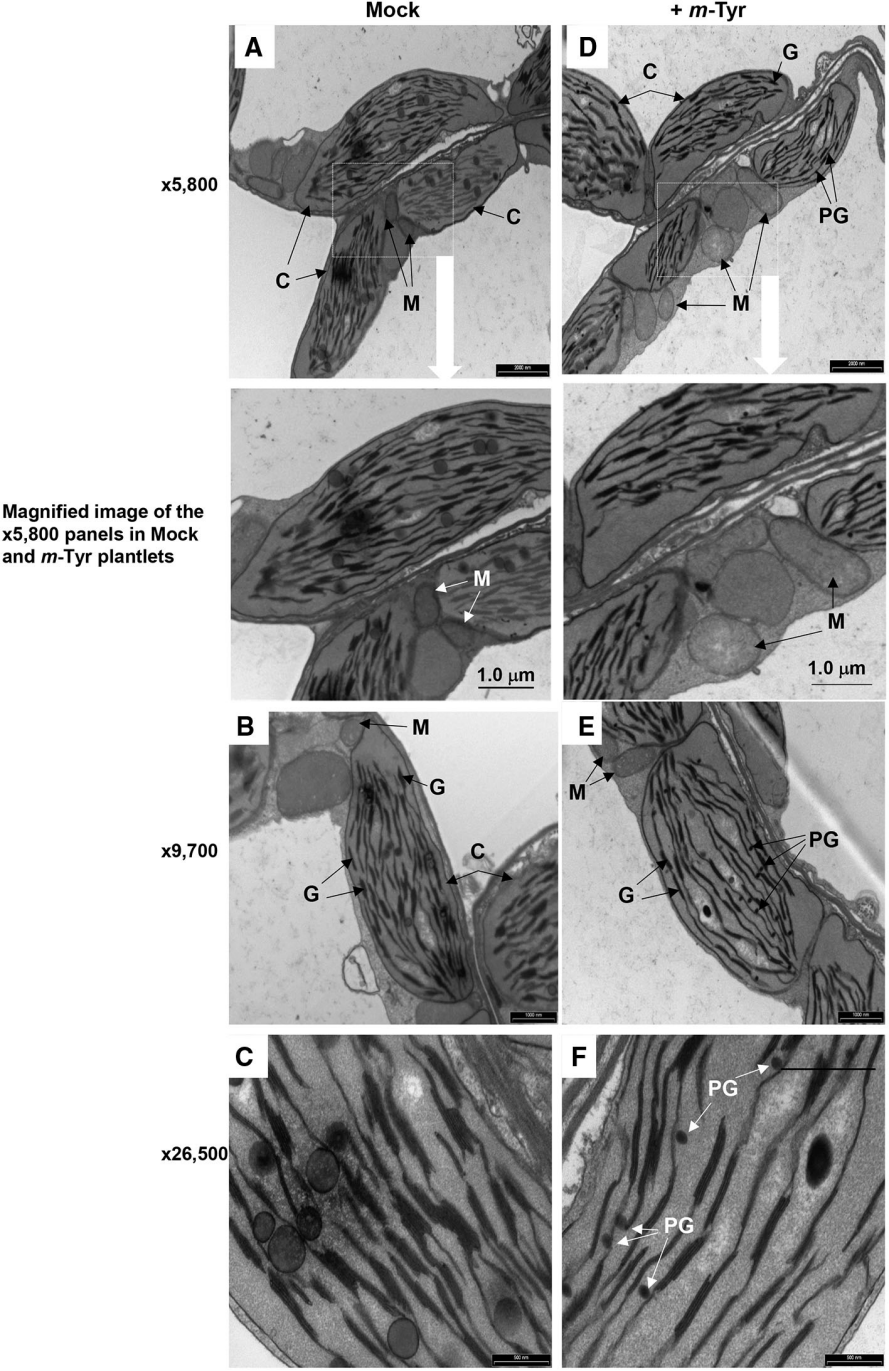
The effect of *m*-tyrosine on organellar morphologies in *Arabidopsis* plants. Representative transmission electron micrographs of ultrathin sections from the hypocotyl tissue of 5-day-old *Arabidopsis* seedlings seeded on Murashige and Skoog (MS)-agar plates in the absence (panels **A**–**C**) or presence of 10 μM *m*-tyrosine (panels **D**–**F**). Mitochondria are labeled by “M,” “C” indicates to chloroplasts, “G” refers to grana stacks, while plastoglobuli are labeled as “PG.” Bars represent 2 µm in panels **A**, **D**, 1.0 µm in panels **B**, **E**, and 0.5 µm in panels **C**, **F**, as indicated in each panel.

*Arabidopsis* seedlings germinated in the presence of *m*-tyrosine (i.e., above 20~40 μM) had yellowish to white cotyledons (**Figure S1**), with reduced chlorophyll content (**Table 2**), thus suggesting that the seedlings are defective in chloroplast development. We observed that the *m*-tyrosine-associated phenotypes can be partially restored by the addition of sucrose to the growth media (**Figure S3**, + sucrose), further suggesting that *m*-tyrosine affects organellar biogenesis during early seedlings establishment and root development. Microscopic analysis of young (i.e., 5-day-old) *Arabidopsis* plantlets treated with 10 μM *m*-tyrosine showed altered chloroplast morphologies, with less grana lamella (**Figure 4**). The relative densities of the thylakoid grana stacks in *m*-tyrosine treated plants were found to be notably lower (0.56 ± 0.07 times) than those of Col-0 plants grown under the same conditions in the absence of *m*-tyrosine (1.00 ± 0.07, **Table 2**). We further noticed the appearance of many plastoglobuli (PG) in the *m*-tyrosine treated plants (**Figures 4D–F**). These lipoprotein particles, which are commonly observed in colored plastids (i.e., chromoplasts), seem particularly prominent in plants affected in chloroplast development (Austin et al., 2006).

**Table 2:** Effects of *m*-tyrosine on protein levels and organellar activities of 5-day-old *Arabidopsis thaliana* (Col-0) seedlings.

*m*-Tyrosine (μM)	Total chlorophyll (μg • gFW^−1^) ^*^1^^	Total protein (mg • gFW^−1^) ^*^1^^	Respiration^*2^ (nmol O_2_ • min^−1^ • gFW^−1^)	Photosynthetic activity (at 150 μE • m^−2^ • s^−2^)^*^3^^	Stacked grana density^*^4^^ (relative pixel intensity)	Mitochondria area^*^5^^ (μm^2^)
0	128 ± 15	2.3 ± 0.18	127.81 ± 7.72	396.02 ± 19.33	1.00 ± 0.04	0.196 ± 0.024
10	119 ± 6.2	1.74 ± 4.21	**80.83 ± 3.51**	**248.32 ± 9.78**	**0.56 ± 0.07**	**0.712 ± 0.165**
20	**112 ± 6.5**	**0.79 ± 1.45**	n.d. ^*^6^^	n.d. ^*^6^^	n.d. ^*6^	n.d. ^*^6^^

*1—The data shows the relative root lengths, number of leaves, total chlorophyll, total protein, respiration, and photosynthetic activities in untreated versus m-tyrosine-treated seedlings. The values are means of 4–7 biological replicates ± SE. Numbers in bold indicate significant differences from Arabidopsis plantlets grown in the absence (0 μM) of m-tyrosine, as determined by Student's t-test (P < 0.05).

*2—Measured by a Clark-type electrode.

*3—Calculated from measurements at different light intensities (**Figure S1**).

*4—The relative membrane densities (i.e., pixel intensities) of grana stacks have been assessed from transmission electron microscopy (TEM) images of ultrathin plant sections (**Figure 4**), using the ImageJ software (Jensen, 2013). Error bars indicate SE (n = 15). Numbers in bold show significant differences from the control (i.e., Arabidopsis plantlets grown in the absence of m-tyrosine), as determined by Student's t-test (P < 0.05).

*5—The average area of mitochondria have been assessed from transmission electron microscopy (TEM) images of ultrathin plant sections (**Figure 4**), using the ImageJ software (Jensen, 2013). Error bars indicate SE (n = 5). Numbers in bold show significant differences from the control (i.e., Arabidopsis plantlets grown in the absence of m-tyrosine), as determined by Student's t-test (P < 0.05).

*6—n.d., not determined.

In addition to altered chloroplast biogenesis defects, we also noticed mild alterations in mitochondria structure in the presence of *m*-tyrosine (i.e., 10 μM). While the electron micrographs of mitochondria of the non-treated plantlets showed characteristic internal cristae formation, as dense folds of the inner-membrane sections (**Figures 4A–C**), many mitochondria in *m*-tyrosine grown seedlings seemed larger (about 3.6x in organellar area, **Table 2**), with reduced inner mitochondrial membrane electron density and less cristae organization (**Figures 4D–F**). Similar organellar morphologies were also noticed in various *Arabidopsis* mutants affected in mitochondria gene expression (see e.g., Keren et al., 2009; Keren et al., 2012; Zmudjak et al., 2013; Cohen et al., 2014).

### *m*-Tyrosine Treated Plants Display Altered Photosynthesis and Respiration Activities

To determine whether the photosynthetic and respiratory activities were altered in plants grown in the presence of *m*-tyrosine, we monitored the O_2_-evolution rates of 5-day-old seedlings in the dark, using a Clark-type electrode (**Table 2** and **Figure S4**). In the dark, the average O_2_-uptake rate of untreated plants was 127.81 ± 7.72 nmol O_2_ ·min^−1^ ·gFW^−1^. *Arabidopsis* seedlings grown in the presence of 10 μM *m*-tyrosine showed lower respiratory activities (i.e., 80.83 ± 3.51 nmol O_2_ ·min^−1^ ·gFW^−1^) than those of the control (i.e., untreated) plants (**Table 2** and **Figure S4**). The photosynthetic activities of control and *m*-tyrosine treated plants were examined by monitoring the O_2_-evolution rates at different light intensities (0–1,000 μmol photons · m^−2^ ·s^−2^). The light compensation point was similar in control plants and seedlings grown in the presence of *m*-tyrosine (12.4 ± 3.7 and 11.2 ± 5.3 µmol ·m^−2^ ·s^−1^) (**Figure S4**). Yet, noticeable differences were observed in light saturation curves between treated (+ *m*-Tyr) and untreated (−*m*-Tyr) plants.

Comparative measurements of light saturation curves of photosynthesis (i.e., O_2_-evolution rates, using a Clark-type electrode) were performed with untreated (**Figure S4**, open boxes) and 5-day-old seedlings grown in the presence of 10 μM *m*-tyrosine (**Figure S4**, filled boxes). The characteristics parameters of the photosynthetic activities were evaluated by fitting the net photosynthesis data to the equation $\left[P\left(I\right)=Pmax\;\left(1-e^{-\alpha \frac{1}{Pmax}}\right)\cdot e^{-\beta \frac{1}{Pmax}}\right]$ (MacIntyre et al., 2002). The maximal photosynthetic yield value (P_max_) of the control plants was 428.5 nmol O_2_ ·min^−1^ ·gFW^−1^, while the P_max_ value of *m*-tyrosine treated plants was notably lower (i.e., 318.4 nmol O_2_ ·min^−1^ ·gFW^−1^). Yet, a lower light intensity was required for the saturation of photosynthesis in the *m*-tyrosine grown plants (i.e., 50.9 µmol ·m^−2^ ·s^−1^, compare with 102.3 µmol ·m^−2^ ·s^−1^ in the control plants). The data also suggested that *m*-tyrosine treated seedlings experienced photoinhibition under lower light intensities, as seen by a decline in O_2_-evolution rates in light intensities higher than ~600 µmol photons · m^−2^ · s^−1^ (**Figure S4**), while the control plants did not show reduced photosynthetic activities in light intensities above ~1,000 µmol photons ·m^−2^ ·s^−1^. Photoinhibition in the *m*-tyrosine treated plants was further apparent by their higher β values (i.e., 0.05198), in comparison to those evaluated for the untreated plants (i.e., β values = 0.009976). These data are indicating that the photosynthetic and respiration activities were reduced in *Arabidopsis* plantlets treated with *m*-tyrosine.

### *Arabidopsis* Plants Treated With *m*-Tyrosine Show Mild Reductions in the Accumulation of Various Organellar Complexes

**Figure 5:**
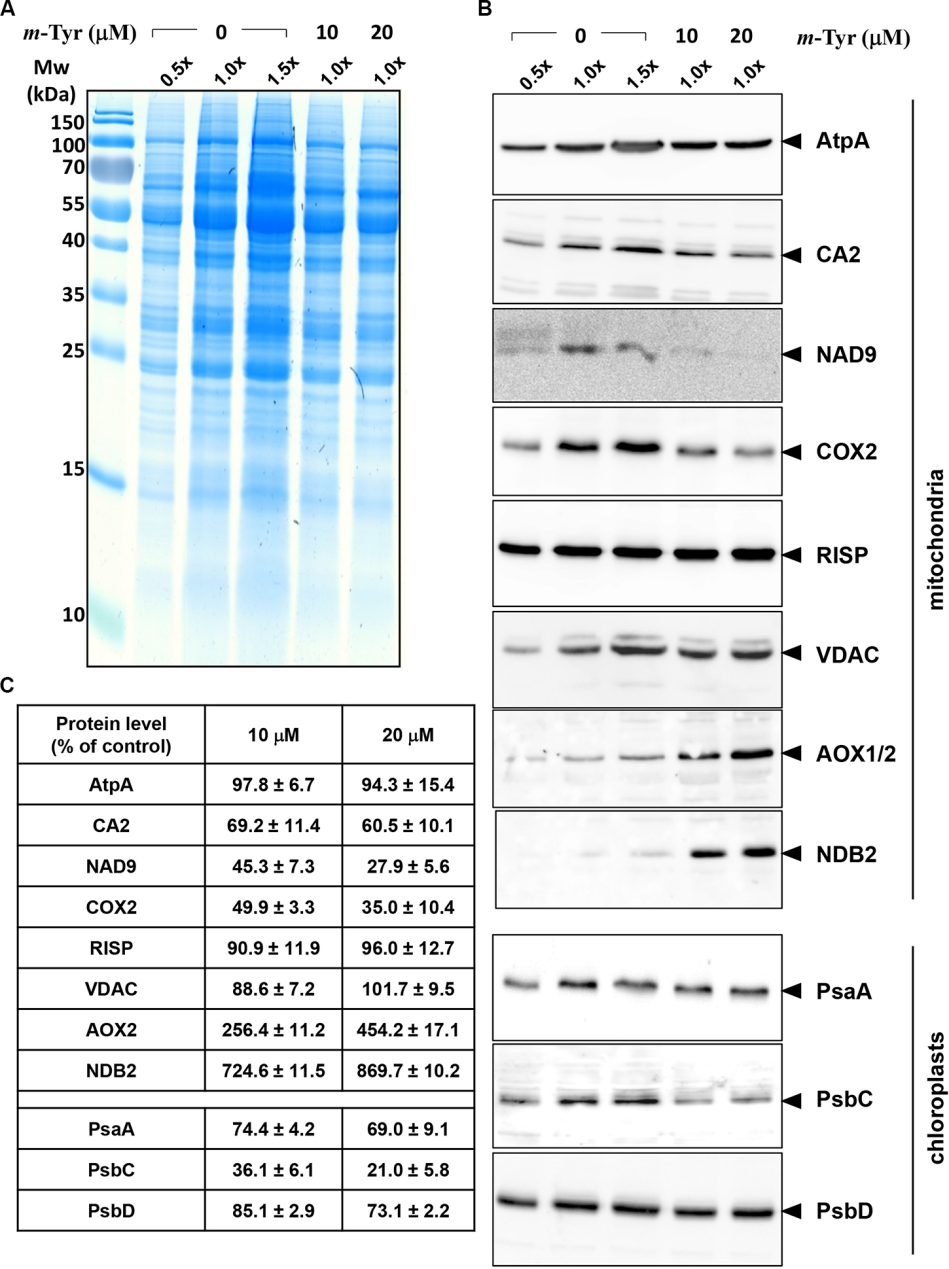
Relative accumulation of organellar proteins in 5-day-old *Arabidopsis* plants grown in the absence or presence of *m*-tyrosine. Immunoblot analyses of 5-day-old *Arabidopsis thaliana* (Col-0) plants grown in the absence or presence of *m*-tyrosine. For the quantification of the relative abundances of organellar proteins in the plants, different amounts of total proteins extracted from control (untreated) and *m*-tyrosine-grown seedlings were loaded and separated by sodium dodecyl sulfate polyacrylamide gel electrophoresis (SDS-PAGE). After electrophoresis, the gel was stained with Coomassie Blue **(A)**, or transferred to a PVDF membrane for immunobloting **(B)**. The blots were probed with polyclonal antibodies raised to different organellar proteins (see **Table S1**), as indicated in the right panel. Detection was carried out by chemiluminescence assays after incubation with horseradish peroxidase (HRP)-conjugated secondary antibody. These include the mitochondrial proteins, ATP-synthase subunit 1 (AtpA), subunit CA2 of complex I (γ-type carbonic anhydrase-like subunit 2) (Perales et al., 2005), and NADH dehydrogenase subunit 9 (NAD9) (Lamattina et al., 1993), the Rieske iron-sulfur protein (RISP) of complex III (cytochrome c reductase) (Carrie et al., 2010), cytochrome oxidase subunit 2 (COX2) of complex IV (C-IV), alternative oxidase (AOX1a) (Elthon et al., 1989), the external NADH-dehydrogenase subunit 2 (NDB2) (Carrie et al., 2008), and the voltage-dependent anion channel (VDAC, or Porin), and the plastidial Photosystem I P700 chlorophyll a apoprotein A1 (PsaA), photosystem II CP43 reaction center protein (PsbC), and photosystem II D2 protein (PsbD). Arrows in the blots indicate to AtpA (55 kDa), CA2 (28 kDa), NAD9 (23 kDa), COX2 (30 kDa), RISP (23 kDa), VDAC (29 kDa), AOX1/2 (36 kDa), NDB2 (50 kDa), PsaA (52 kDa), PsbC (43 kDa), PsbD (39 kDa). Hybridization signals were analyzed by chemiluminescence assays after incubation with HRP-conjugated secondary antibody. The intensities of protein signals in panel **B** were evaluated using the ImageJ software (Jensen, 2013). The data shows the relative accumulation of each protein in untreated *versus m*-tyrosine-treated seedlings (panel **C**). The values are means of three biological replicates ± SE.

The accumulation of organellar proteins was analyzed in 5-day-old *Arabidopsis* seedlings treated with 10 or 20 μM *m*-tyrosine. Total protein, extracted from control (−*m*-Tyr) and *m*-tyrosine treated plantlets, was analyzed by (12%) SDS-PAGE, following immunoblotting with antibodies raised against various organellar proteins (**Figure 5A** and **Table S1**). The steady-state levels of the mitochondrial NAD9, CA2, and COX2 (i.e., between 30 up to 55% reductions), the plastidial PsbC protein (about 64% lower), and to a lesser extent the plastidial PsaA subunit (~25% lower), were all found to be reduced in seedlings germinated in the presence of *m*-tyrosine (**Figures 5B, C**). The accumulations of AtpA, RISP, VDAC, and PsbD subunits were not significantly affected by the addition of *m*-tyrosine to the growth media (**Figures 5B, C**). The rotenone-insensitive NADH dehydrogenases (NDBs) and alternative oxidases (AOXs) are induced in a coordinated manner under respiratory chain dysfunction and oxidative stress conditions (Clifton et al., 2005; Garmier et al., 2008; Rasmusson et al., 2008; Yoshida and Noguchi, 2009; Keren et al., 2012). The signals of AOX and NDB2 were both found to be somewhat higher (about 2.5 and 7.2 times higher, respectively) in the 5-day-old *m*-tyrosine treated *Arabidopsis* plantlets (**Figures 5B, C**).

**Figure 6:**
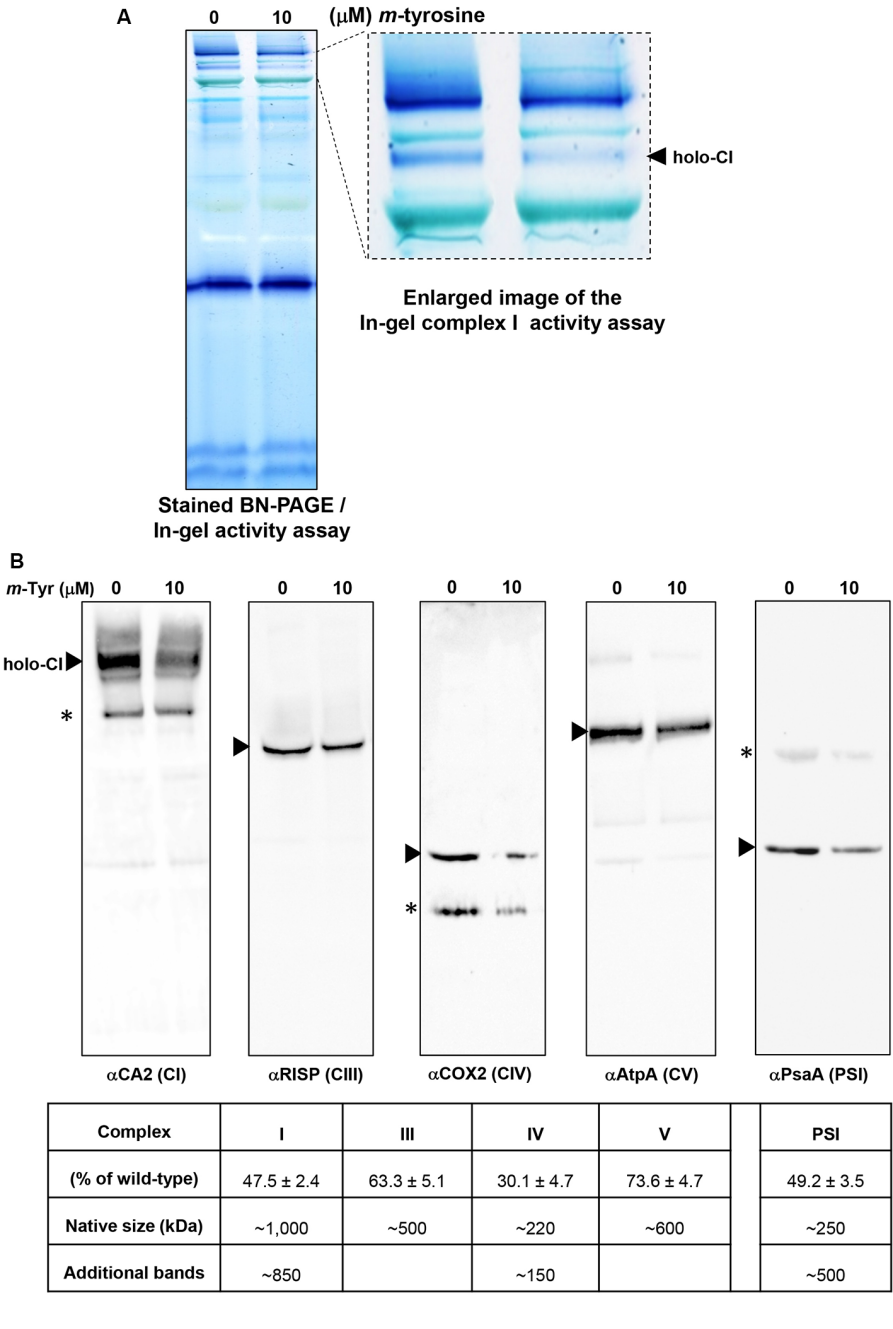
Relative accumulation of native organellar complexes in 5-day-old *Arabidopsis* plants grown in the absence or presence of *m*-tyrosine. Blue native-polyacrylamide gel electrophoresis (BN-PAGE) of crude organellar preparations was performed according to the method described in Pineau et al. (2008). Equal amounts of crude membranous fractions (equivalent to 50 mg fresh-weight seedlings), were obtained from 5-day-old *Arabidopsis* seedlings, solubilized with 1.5% n-dodecyl-β-d-maltoside (DDM), and the organellar complexes were resolved by BN-PAGE. (Panel **A**) shows stained PAGE, followed by in-gel activity assay of complex I, according to the method described in Eubel et al. (2005). For immunodetections (panel **B**), proteins were transferred from the native gels onto a polyvinylidene difluoride (PVDF) membrane (see *Materials and Methods*) and then probed with specific antibodies (see **Table S1**), as indicated below each blot. Arrows indicate to the native mitochondrial complexes I (~1,000 kDa), III (dimer, ~500 kDa), IV (~220 kDa), V (~600 kDa), and the plastidial PSI complex (~250 kDa). Asterisks in the COX2 and PsaA panels indicate the presence of lower or higher (respectively) molecular weight particles of unknown origins. Hybridization signals were analyzed by chemiluminescence assays after incubation with horseradish peroxidase (HRP)-conjugated secondary antibody. The intensities of protein signals in (panel **B**) were evaluated using the ImageJ software (Jensen, 2013). The data shows the relative accumulation of each protein in untreated *versus m*-tyrosine-treated seedlings. The data shows the relative accumulation of each protein in untreated versus m-tyrosine-treated seedlings (panel **C**). The values are means of three biological replicates ± SE.

To investigate the effects of *m*-tyrosine on the accumulation of native organellar complexes, crude membranous fractions were obtained from 5-day-old seedlings germinated in the absence or presence of 10 μM *m*-tyrosine. The membranous proteins were separated under native conditions by blue native-polyacrylamide gel electrophoresis (BN-PAGE), and then subjected to immunoblot analyses with various antibodies (**Table S1**), as indicated in **Figure 6**. Arrows indicate to the native CI, CIII, VIV, CV, and PSI complexes. Under the gel electrophoretic conditions (Pineau et al., 2008; Shevtsov et al., 2018), we noticed to reduced steady-state levels of the respiratory complexes CI (47.5%), CIII (36.7%), CIV (59.9%), and CV (26.4%) upon *m*-tyrosine treatment (**Figure 6**). Reduced CI activity was also apparent by “*in-gel*” activity assays (**Figure 6A**). Similarly, immunoblots with PsaA indicated to reduced accumulation in photosystem I (PI) in seedlings grown in the presence of 10 μM *m*-tyrosine (**Figure 6B**). The immunoassays with anti-CA2, COX2, and anti-PsaA antibodies further indicated the presence of additional protein bands (**Figure 6B**, marked with asterisks). We speculate that the lower molecular weight bands in the CA2 and COX2 blots (i.e., about 800 and 150 kDa, respectively) may correspond to a partially assembled sub-CI and CIV particles, respectively (Meyer et al., 2011), whereas the upper-band (~500 kDa) in the PsaA blot may correspond to an oligomeric state of PSI (Salomon and Keren, 2011).

### Analysis of *m*-Tyrosine Incorporation Into the Plant Proteome

The Phe-analog, *m*-tyrosine, affects early seedlings development and radicle elongation of plants (**Figures S1** and **S3**, and Bertin et al., 2007). Growing *Arabidopsis* seedlings in the presence of *m*-tyrosine results with a reduction in the level of free-Phe (**Figure 1**). Accordingly, *adt* mutants are more severely affected by *m*-tyrosine (**Figures 3** and **S2**), whereas the *adt2-1D* mutant plant-line that accumulates higher levels of free-Phe (Cho et al., 2007) seems less susceptible to *m*-tyrosine toxicity (Huang et al., 2010). Similarly, the addition of exogenous Phe can partially restore the growth and developmental defect phenotypes associated with *m*-tyrosine (**Figure 2**, and Bertin et al., 2007). Here, we further show that *m*-tyrosine alters the biogenesis and activities of both the mitochondria and chloroplasts during early developmental stages (**Figures 4**, **6**, **S1**, and **S3**, **Table 2**). It remains possible, therefore, that the developmental defect phenotypes cause by *m*-tyrosine may relate to its misincorporation to the mitochondrial and plastidial proteomes.

To analyze whether *m*-tyrosine is incorporated into *Arabidopsis* proteins, we performed MS-based proteomic analyses of 5-day-old plantlets grown in the absence or presence of *m*-tyrosine (**Supplemental Table S3**, doi: 10.6084/m9.figshare.11627211). The proteomic analyses indicated many Phe → *m*-Tyr/Tyr exchanges (i.e., due to their identical masses it is not possible to distinguish between the native amino acid Tyr and its structural related isomers, *o-* and *p-* and *m-*tyrosine in the MS data) in various proteins of *Arabidopsis* seedlings grown in the presence of *m*-tyrosine, which were not observed in plants grown in the absence of the analog (i.e., under the same growth conditions). **Table 3** summarizes the MS-based data of four independent proteomic analyses of 5-day-old seedlings grown in the absence (control) or presence of 10 μM and 20 μM *m*-tyrosine. The frequencies of Phe mistranslation is dose-dependent, as the misincorporation frequencies increase about two folds when plants were treated with 20 μM instead of 10 μM *m*-tyrosine (**Table 3**). In accordance with previous observations suggesting that organellar PheRSs might be more prone to misacylation of *m*-tyrosine (Klipcan et al., 2009; Klipcan et al., 2010), the proteomic data indicated that Phe → *m*-Tyr/Tyr exchanges occur more frequently during the synthesis of organellar proteins, and in particularly of those translated within the chloroplasts (**Table 3** and **Supplementary Data File 1**).

**Table 3:** Summary of four independent proteome analyses of enriched organellar fractions obtained from 5-day-old seedlings grown in the absence or presence of *m*-tyrosine.

	Protein I.D.			*m*-Tyrosine (μM)		
				0	10	20
	Protein	Locus	Loci of synthesis*^2^	No. of Phe → *m*-Tyr replacements*^1^		
**Plastid**	ATP synthase subunit beta	AtCg00480	C	0	3-4	5
	Ribulose bisphosphate carboxylase large chain	AtCg00490	C	0~1	2~4	2~6
	ATP synthase subunit alpha	AtCg00120	C	0	1~2	2
	Photosystem II CP47 reaction center protein	AtCg00680	C	0	2~4	4
	Photosystem II D2 protein	AtCg00270	C	0	0~1	1~3
	Chlorophyll a-b binding protein 3 LHCB3	At5g54270	Cyt	0	0~1	0~2
	Chlorophyll a-b binding protein 2, LHCB2.2	At2g05070	Cyt	0	0~1	0~2
	Cytochrome b559 subunit alpha PsbF	AtCg00570	C	0	0~1	0~2
	Cytochrome f PetA	AtCg00540	C	0	0~1	0~2
**Mitochondria**	ADP, ATP carrier protein 1	At3g08580	Cyt	0	1~4	3~4
	ATP synthase subunit beta-1	At5g08670	M	0	0	0~3
	ATP synthase subunit beta-2	At5g08690	M	0	0	0~3
	Mitochondrial outer membrane protein porin 3	At5g15090	Cyt	0	0	0~2
	NADH dehydrogenase subunit 9	AtMg00070	M	0	0	0~2
	ATP synthase subunit alpha, mitochondrial	At2g07741	M	0	5~7	6~7
**Other**	Beta-glucosidase	At3g09260	Cyt	0	3	1~7
	Inactive GDSL esterase/lipase-like protein 23	At1g54010	Cyt	0	0~2	0~3
	PYK10-binding protein 1	At5g16420	Cyt	0	0~1	2

*1—LC-MS/MS data of 4–6 independent repeats.

*2—C, chloroplasts; Cyt, cytosol; M, mitochondria.

### Mutants Affected in FtsH2 Show Hypersensitivity to *m*-Tyrosine

Our results (**Table 3**) indicate that *m*-tyrosine can be delivered into the proteomes of plants, an abnormal cellular condition that can be associated with increased protein turnover (Rodgers et al., 2002). FtsH is a membrane-bound ATP-dependent zinc metalloprotease complex, which has been characterized in bacteria and organelles of eukaryotic cells (Kato and Sakamoto, 2018 and references therein). While *Escherichia coli* harbors a single *FtsH* gene, *Arabidopsis* plants encode 12 related FtsHs that play key roles in protein quality control, as removal of damaged and abnormal proteins (Ostersetzer and Adam, 1997; Adam and Ostersetzer, 2001; Zaltsman et al., 2005; Sakamoto, 2006). Nine FtsHs reside in the plastids, whereas the other three paralogs are located within the mitochondria (Kato and Sakamoto, 2018). In chloroplasts, FtsH is made of different hetero-hexamer complexes (Moldavski et al., 2012). FtsH2 (At2g30950, also denoted as VAR2) is the most abundant isoform within the plastids. *var2* mutants show aberrant leaf development with severe variegation phenotypes, which are related to altered chloroplast development (Chen et al., 2000; Takechi et al., 2000; Sakamoto et al., 2003).

**Figure 7:**
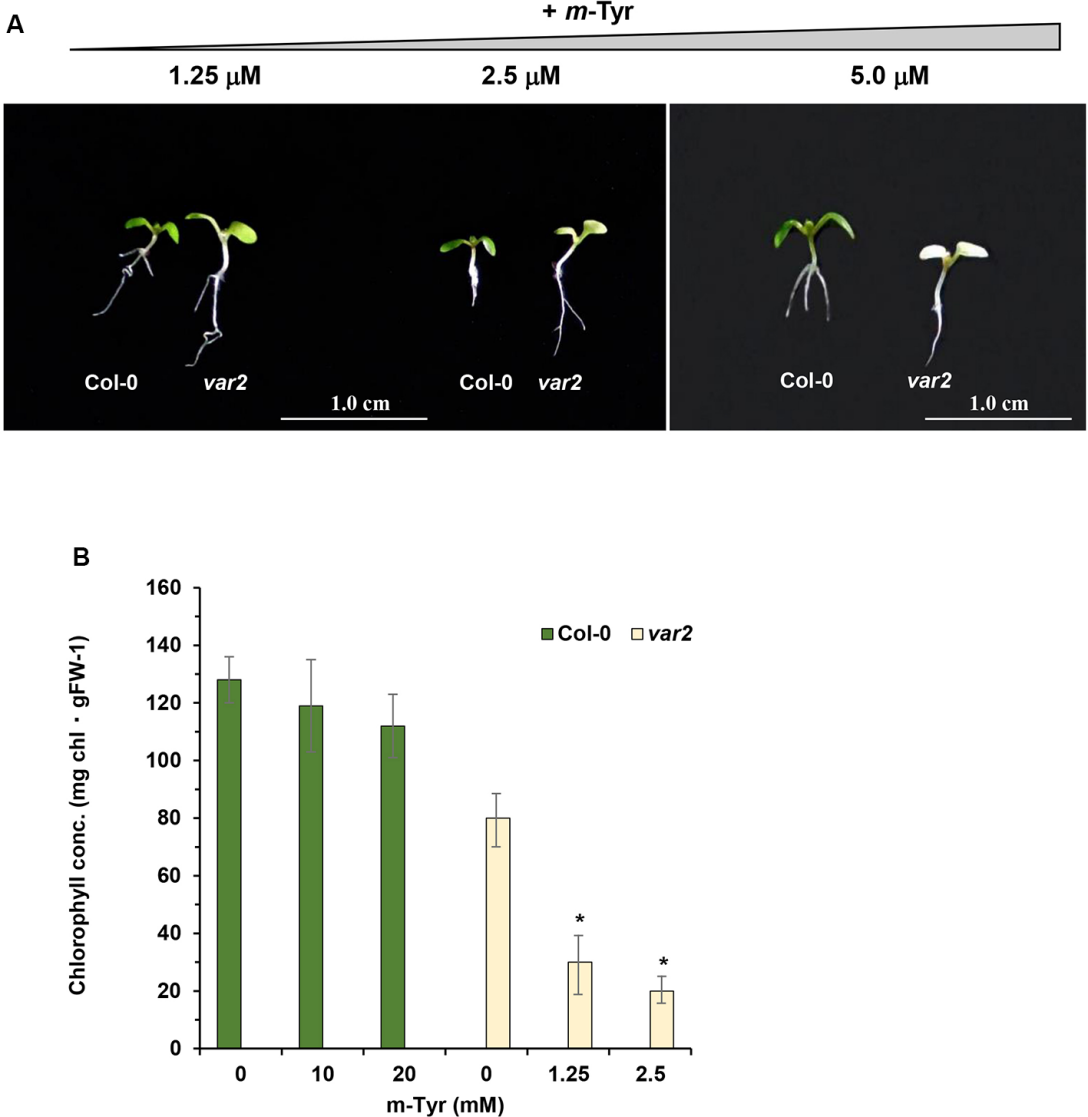
The effect of *m*-tyrosine on *Arabidopsis* wild-type (Col-0) and *var2* knockout mutant-line. **(A)** Seeds of *Arabidopsis* wild-type (Col-0) and homozygous *var2* mutant-line (Takechi et al., 2000; Sakamoto et al., 2003; Zaltsman et al., 2005) (generously provided by the group of Prof. Zach Adam, The Hebrew University) were germinated in Murashige and Skoog (MS)-agar plates in the absence or presence of different concentration of *m*-tyrosine. The values are means of three biological replicates with about 25 seedlings, as indicated in the panel. The figure shows 5-day-old seedlings. Bars represent 1 cm in each panel. The values are means of three biological replicates with about 25 seedlings (i.e., 5-day-old). Error bars indicate one standard deviation. Asterisk in (panel **B**) indicates a significant difference from *var2* mutants control plants (Student's T-test, P ≤ 0.05).

Based on these data we speculated that *var2* mutants might show higher sensitivity to *m*-tyrosine, due to the accumulation of abnormal and damaged proteins within the plastids. To examine this hypothesis, we analyzed the root development and early seedling establishment of *Arabidopsis* wild-type (Col-0) and *var2* mutants (Takechi et al., 2000; Sakamoto et al., 2003; Zaltsman et al., 2005), germinated in the absence or presence of various concentrations of *m*-tyrosine. The data indicated that *var2* mutants seem to be more susceptible to inhibition by *m*-tyrosine (**Figure 7**). In particular, the Phe-analog, *m*-tyrosine, had a strong effect on chloroplast biogenesis of *var2* plants, as evident by the cotyledon chlorosis (**Figure 7A**) and reduced chlorophyll content (**Figure 7B**). Reduced leaf pigmentation in *var2* was already observed at *m*-tyrosine concentrations ≥ 1 μM, while no chlorophyll was detected when the mutants were grown in *m*-tyrosine concentrations above 5.0 μM (**Figure 7**).

### Mutants Affected in Autophagy Show Higher Sensitivity to *m*-Tyrosine Toxicity

Autophagy is an essential process in eukaryotic cells that involves a control degradation of large substrates including protein aggregates, pathogens, and organelles (reviewed by e.g., Avin-Wittenberg et al., 2018). Forward genetics led to the identification of many autophagy-related loci in *Arabidopsis*, denoted as *ATG* genes (Young et al., 2019). Among these genes are *ATG5* (At5g17290) and *ATG7* (At5g45900), which their mutants show a full inhibition of autophagy (Barros et al., 2017 and references herein). When *Arabidopsis* wild-type plants (Col-0) and *atg* mutants (Hofius et al., 2009; Yoshimoto et al., 2009; Avin-Wittenberg et al., 2015) were germinated in the absence or presence of various concentrations of *m*-tyrosine (**Figure S5A**), the *atg* mutant-lines were found to be more susceptible to high concentrations of *m*-tyrosine (i.e., > 10 μM). Yet, the phenotypes of the autophagy mutants were undistinguishable from those of wild-type (Col-0) plants at lower concentrations of the Phe-analog (**Figure S5B**). These results seem to be correlated with the organellar biogenesis defects of *Arabidopsis* plantlets germinated in the presence of *m*-tyrosine (**Figures 4**, **S1**, and **S3**, **Table 2**).

## Discussion

### *m*-Tyrosine Is a Strong Allelochemical That Affects Plants Early Growth and Development

*m*-Tyrosine is a non-proteinogenic analog of the aromatic amino acids phenylalanine and tyrosine, which acts as a natural herbicide, affecting the post-germination development and early establishment of nearby plant life (Bertin et al., 2007). Published data indicates that *m*-tyrosine is significantly more toxic to plants than its structural related isomers*, o-* and *p-*tyrosine (Bertin et al., 2007). Our data provide further insights into *m*-tyrosine-mediated phytotoxicity. Under different conditions, and more particularly under oxidative stress, *m*-tyrosine is accumulating to higher levels in the cell (Ipson and Fisher, 2016). *m*-tyrosine is directly generated by hydroxyl radical attack of phenylalanine when levels of reactive oxygen species are elevated (Rodgers and Shiozawa, 2008), or might be produced enzymatically, as indicated in a few plant species (Mothes et al., 1964; Bertin et al., 2007; Huang et al., 2012). In *F. rubra*, *m*-tyrosine biosynthesis occurs through the hydroxylation of phenylalanine in the root tips. The allelochemical is then released to the biosphere, were it inhibits the post-germination development of nearby plants (Huang et al., 2012).

*Arabidopsis* seedlings germinated in the presence of *m*-tyrosine show slow seedlings establishment, altered root elongation, and reduced biomass (**Figures 3**, **S1**–**S3**, and **S5**, and Bertin et al., 2007). The severity of these phenotypes are correlated with the levels of *m*-tyrosine in the growth media, with calculated IC_50_ value of 2.365 μM (**Figure S1**). At *m*-tyrosine concentrations higher than 40~80 μM, the plants show post-germination arrest of embryo development (**Figure S1**). Apparently, while *m*-tyrosine had no obvious effect on the photosynthetic activity of lettuce seedlings (Bertin et al., 2007), at concentrations above 40 μM *Arabidopsis* seedlings showed signs of leaf (i.e., cotyledon) chlorosis (**Figure S1**). Analyses of 5 day-old seedlings grown in the absence or presence of 10 μM *m*-tyrosine indicated to reduced chlorophyll production (**Table 2**), altered organellar biogenesis (**Figure 4**) and reduced photosynthetic activities (**Table 2** and **Figure S4**). Our data also indicate to a reduction in oxygen consumption when the plants are grown in the presence of *m*-tyrosine (**Table 2**). Respiration in some CI-deficient *Arabidopsis* mutants can be upregulated, a phenomenon that has been associated with the induction of the alternative respiratory pathway, mediated by non-phosphorylating NAD(P)H dehydrogenases (NDs) and alternative oxidases (AOXs) (see e.g., Kuhn et al., 2015). We speculate that although AOX and ND2 are upregulated in *m*-tyrosine treated plants, the high energy demand (i.e., ATP production) during germination and early seedling metabolism necessitates the electron transport *via* the canonical respirasome mediated pathway. Accordingly, in different plant species, the affinity of AOX is significantly lower compared to that of COX to O_2_ (see e.g., van Dongen et al., 2011). The *m*-tyrosine-mediated defect phenotypes can be partially rescued by the addition of sucrose to the growth media (**Figure S3**), in a similar manner to the effects of sugar on *Arabidopsis* mutants affected in chloroplast or mitochondria biogenesis (see e.g., Keren et al., 2012; Van Dingenen et al., 2016).

Based on the experimental results and published data we consider two different cellular pathways in which *m*-tyrosine inhibits the early establishment of *Arabidopsis* plants. Together, the analyses of the steady-state levels of free amino acids in control and *m*-tyrosine-grown seedlings (**Figure 1** and **Table S2**), the effects of various amino acids added to the growth media on *m*-tyrosine toxicity (**Figure 2**), the higher-sensitivity of *adt* mutants to *m*-tyrosine (**Figures 3** and **S2**) and lower sensitivity of *adt2-1D* mutant which accumulates higher levels of Phe (Cho et al., 2007), strongly suggest that *m*-tyrosine affects the biosynthesis of phenylalanine in early-developing *Arabidopsis* seedlings. Another molecular mechanism by which *m*-tyrosine can affect *Arabidopsis* vitality involves the misincorporation of this aromatic non-proteinogenic analog into the plant proteome instead of Phe, and in particular to various organellar proteins. These are evident by proteomic data (**Table 3** and **Supplemental Table S3**, doi: 10.6084/m9.figshare.11627211), altered organellar biogenesis and functions (**Table 2**, **Figures 4**–**6** and **S4**), the hypersensitivity of *var2* mutants to *m*-tyrosine (**Figure 7**), as well as previous data indicating that tRNA^Phe^ can be charged with *m*-tyrosine, *in vitro* (Gurer-Orhan et al., 2006; Klipcan et al., 2009).

### Phenylalanine Biosynthesis Is Affected in *Arabidopsis* Plants Treated With *m*-Tyrosine

Published data (Huang et al., 2010; Höhner et al., 2018) and our own results (**Figures 2** and **S2**) strongly suggest that *m*-tyrosine toxicity is affected by the (bio-) availability of Phe. Accordingly, exogenous Phe can partially rescue the growth defect phenotypes mediated by *m*-tyrosine (**Figure 2**). Seedlings grown in the presence of the amino acid methionine (Met) are also less susceptible to inhibition by *m*-tyrosine (**Figure 2**, and Bertin et al., 2007). Therefore, it remains possible that Met plays a non-redundant role through indirectly affecting Phe metabolism, or by scavenging reactive oxygen species (ROS) that may accumulate to higher levels in the *m*-tyrosine treated plants. Methionine may also act as catalytic antioxidants, protecting protein where they reside, as well as other molecules within the cells (Luo and Levine, 2009). Accordingly, *Arabidopsis* seedlings germinated in the presence of *m*-tyrosine show altered organellar activities (**Table 2** and **Figure S4**) and accumulate high levels of AOX (**Figure 5**).

An effect of *m*-tyrosine on Phe biosynthesis is further supported by genetic analyses. Arogenate dehydratase (ADT) is a key enzyme in Phe biosynthesis, converting arogenate into Phe. ADT activity in plants is positively regulated by Tyr and negatively regulated by Phe (Jung et al., 1986; Siehl and Conn, 1988). While *Arabidopsis adt* mutants show a hipper sensitivity to *m*-tyrosine (**Figures 3** and **S2**), the *adt2-1D* mutant-line that accumulates higher levels of Phe, most likely due to a disruption in the feedback inhibition (i.e., allosteric effects) of the ADT enzyme (Cho et al., 2007), are less susceptible to *m*-tyrosine toxicity (Huang et al., 2010). Accordingly, analyses of the steady-state levels of free amino-acids indicate that the phytotoxic effects of *m*-tyrosine correlate with altered amino acid metabolism, in particularly phenylalanine (**Figure 1** and **Table S2**). Taken together, these data suggest that *m*-tyrosine reduces the biosynthesis of Phe, probably by directly affecting the activity of ADT. However, these assumptions need to be validated experimentally.

### Misincorporation of Free *m*-Tyrosine Into the Proteome of *Arabidopsis* Plants

Another molecular mechanism, by which *m*-tyrosine affects plants, may involve the incorporation of unnatural chemical groups (i.e., non-canonical amino acids) into proteins (Smith and Fowden, 1968), which are expected to affect the activities of proteins and enzymes, or to interfere with the functions of native side chains. Proteomic analyses of plants germinated in the absence or presence of *m*-tyrosine indicate that this analog is misincorporated into the plant proteome (**Table 3**, and **Supplemental Table S3**, doi: 10.6084/m9.figshare.11627211). These data are also in agreement with earlier reports suggesting that *m*-tyrosine may be wrongly delivered into proteins in bacteria (Aronson and Wermus, 1965) and animals (Gurer-Orhan et al., 2006). In *Arabidopsis*, the majority of the Phe → *m*-Tyr/Tyr replacements are identified in proteins that are expressed within the organelles, in particular plastidial ones (**Table 3**). Furthermore, analyses of mutant plants affected in FtsH2, a major component of the chloroplast protein quality control system (Kato and Sakamoto, 2018), resulted with an increased sensitivity to *m*-tyrosine, an effect that is expected to be associated with the accumulation of damaged and abnormal organellar proteins (Ostersetzer and Adam, 1997; Adam and Ostersetzer, 2001; Sakamoto, 2006). Also, mutant plants affected in autophagy are more severely affected in seedling establishment and show strong cotyledon bleaching at *m*-tyrosine concentrations above 10 μM (**Figure S5**), although the effects at high *m*-tyrosine could rather relate to general stress responses rather than to a specific cellular damage.

How can *m*-tyrosine be incorporated into the plant proteome? Aminoacyl tRNA synthases (aaRSs) ensure the integrity of the translation of the genetic code, by covalently attaching an appropriate amino acid to the corresponding nucleic acid adaptor tRNA molecule. Although aaRS are known to be highly specific, mistakes in the recognition may still occur, due to stereo-chemical similarities shared by some native amino acids and their non-proteinogenic analogs (Moghal et al., 2014; Chaliotis et al., 2016; Mohler and Ibba, 2017). A key repair mechanism to ensure the acetylation of the correct amino acid involves an intrinsic editing activity, by which misacylated tRNAs can be hydrolyzed. In plants, the attachment of phenylalanine to tRNA^Phe^ is catalyzed by two major forms of PheRSs. These include i) a heterotetrameric (2·αβ) form, which is structurally related to the bacterial 2·αβ enzymes and acts in the cytosol, and ii) a degenerated monomeric form (contains fragments of α and β), which dually functions in the plastid and mitochondria of plants (Duchêne et al., 2005; Klipcan et al., 2010).

The aromatic amino acids Phe, Tyr, and their cognate non-proteinogenic *m*-tyrosine analog, are distinguished by a single hydroxyl group at the aromatic ring, and thus discrimination between these molecules may not always be accurate (Roy et al., 2004; Kotik-Kogan et al., 2005; Ling et al., 2007; Klipcan et al., 2009; Moghal et al., 2014; Popp et al., 2015). To maintain accuracy during translation, the PheRS enzyme possess an editing domain that hydrolyzes misacylated tRNA^Phe^ (Roy et al., 2004; Kotik-Kogan et al., 2005; Ling et al., 2007). Structural and biochemical assays suggest that bacterial and both eukaryotic PheRS forms can load *m*-tyrosine into tRNA^Phe^ (Klipcan et al., 2009; Moghal et al., 2016). However, only the bacterial enzyme seems to be able to efficiently hydrolyze the misacylated *m-*tyrosine (Klipcan et al., 2009). In the cytosolic PheRS form of eukaryotes, discrimination against *m*-tyrosine mainly occurs during the aminoacylation step (Klipcan et al., 2009). Structural and biochemical studies suggest that organellar PheRS is not able to discriminate against *m*-tyrosine and lacks editing activity (Klipcan et al., 2009; Klipcan et al., 2010). It is, therefore, expected that the dually-localized organellar PheRS enzyme will be more prone to errors, opening up the rout for delivery of misacylated tRNA^Phe^-*m*-tyrosine to the organellar ribosomes.

### Why Are Plants More Sensitive to *m*-Tyrosine Than Other Life Forms?

Currently, we are not able to provide with a definitive answer. Plants are highly susceptible to *m*-tyrosine [**Figures S1** and **S3**, and (Bertin et al., 2007)], with IC_50_ (i.e., root growth inhibition) at the low micromolar range (**Figure S1**), about 100~1,000 times more toxic to plants than to bacterial (Smith et al., 1964; Aronson and Wermus, 1965), yeast (Bullwinkle et al., 2014), or animal cells (Gurer-Orhan et al., 2006). Phe is an essential α-amino acid that is produced through the shikimate pathway, which is present in bacteria, fungi, and plants, as well as in some protozoa, but not in Animalia who need to obtain aromatic amino acids from their diet. While animals cannot produce Phe, it was previously shown that *m*-tyrosine can be biodegraded in several microorganisms, which can utilize this metabolite as the sole source of carbon, nitrogen, and energy (Khan et al., 2013 and references therein). In addition, the levels of free Phe, seem to be notably higher in animal cells (about 55 to 60 μM in human cells) then in plants (i.e., *Arabidopsis*, ~15 μM) (Kaufman, 1999; Voll et al., 2004).

As indicated above, there are also differences in the way that *m*-tyrosine can be delivered into the proteomes of different organisms. While prokaryotes encode a single heterotetrameric PheRS that is able to efficiently hydrolyze the misacylated *m-*tyrosine (Klipcan et al., 2009), eukaryotes possess two PheRS isoforms, one of which resides within the organelles (Klipcan et al., 2009). In plants, the organellar PheRS isoform resides in both the plastids and mitochondria (Duchêne et al., 2005). Plastid biogenesis seems in particularly amenable to *m*-tyrosine toxicity. This is apparent by altered chloroplast morphologies, reduced grana stacking, the presence of many plastoglobuli (**Figure 4**) and lower photosynthetic rates (**Table 2** and **Figure S4**), associated with *Arabidopsis* plantlets germinated in the presence of *m*-tyrosine, as well as the fact that many polypeptides that undergo Phe → (*m*-)Tyr exchanges are identified as plastidial proteins (**Table 3**). These may relate to the high complexity of the translational apparatus in plant chloroplasts (Zoschke and Bock, 2018). We speculate that the effects of *m*-tyrosine on Phe biosynthesis together with the ability of the organellar PheRS to deliver the non-proteinogenic analog to both mitochondrial and plastidial proteins makes plants highly sensitive to *m*-tyrosine.

In summary, this work is founded on earlier reports that indicate that *m*-tyrosine is highly toxic to different plant species. Based on the data we consider two related molecular mechanisms by which *m*-tyrosine can affect angiosperm's root development, radicle elongation, and early establishment. These involve (*a*) a direct interference in the metabolism of various amino acids, and in particular Phe, and (*b*) the misincorporation of the Phe-analogue to some cytosolic and mainly the plant organellar proteins. The correct transfer of information from the genome to proteins is pivotal for the development and physiology of plants, as well as for other organisms. The specificity of aminoacyl-tRNA synthetases (aaRSs) is key for ensuring the proper decoding of the genetic information into proteins. Distinguishing between closely related amino acids and their analogs by aaRSs is not always accurate, leading to errors in tRNA loading and hence for the translation of aberrant polypeptides. When a non-cognate amino acid is activated, some aaRSs employ an editing mechanism leading to the hydrolysis of the misacylated tRNA molecule. Biochemical and structural studies suggest that organellar PheRS forms lack editing activity. The two effects (i.e., altered Phe biosynthesis and increased misincorporation of *m*-tyrosine) are related to one another, i.e., reduced availability of the natural amino acid Phe would ultimately result an increased misincorporation of its analogous compound, *m*-tyrosine, in particularly into organellar proteins (Klipcan et al., 2009). The effects of *m*-tyrosine are in particularly prevalent during early development, this in accordance with mutant plants affected in organellar biogenesis, which also show early growth retardation, inhibition of root development, and altered seedling establishment phenotypes (see e.g., Keren et al., 2012; Van Dingenen et al., 2016).

## Data Availability Statement

The datasets generated for this study can be found in the https://doi.org/10.6084/m9.figshare.11627211.

## Author Contributions

HZ: Experimental design, plant growth and analysis, analyses of the protein profiles, BN-PAGE assays. NM: Assisted in growth and analyses: HM and TA-W: GC-MS analyses of plant extracts. LK: Assisted with experimental design, co-corresponding author. OO-B: Principal Investigator, MS preparation and corresponding authors.

## Funding

Research at the TA-W group is funded by the Israel Science Foundation (ISF grant no. 1899/16). This work was supported by grants from the ‘Israel Ministry of Agriculture, Nitsan Fund' (No. 20-01-0162) to LK and OO-B and the ‘Israel Science Foundation' (ISF grant no. 741/15) to OO-B.

## Conflict of Interest

The authors declare that the research was conducted in the absence of any commercial or financial relationships that could be construed as a potential conflict of interest.
