# Supplementary material for: The Phytotoxicity of Meta-Tyrosine Is Associated With Altered Phenylalanine Metabolism and Misincorporation of This Non-Proteinogenic Phe-Analog to the Plant's Proteome
Source: Front Plant Sci. 2020 Mar 6;11:140. doi: 10.3389/fpls.2020.00140 (PMC7069529; doi:10.3389/fpls.2020.00140)
Supplement: Supplementary file 1 [file DataSheet_1.pdf]

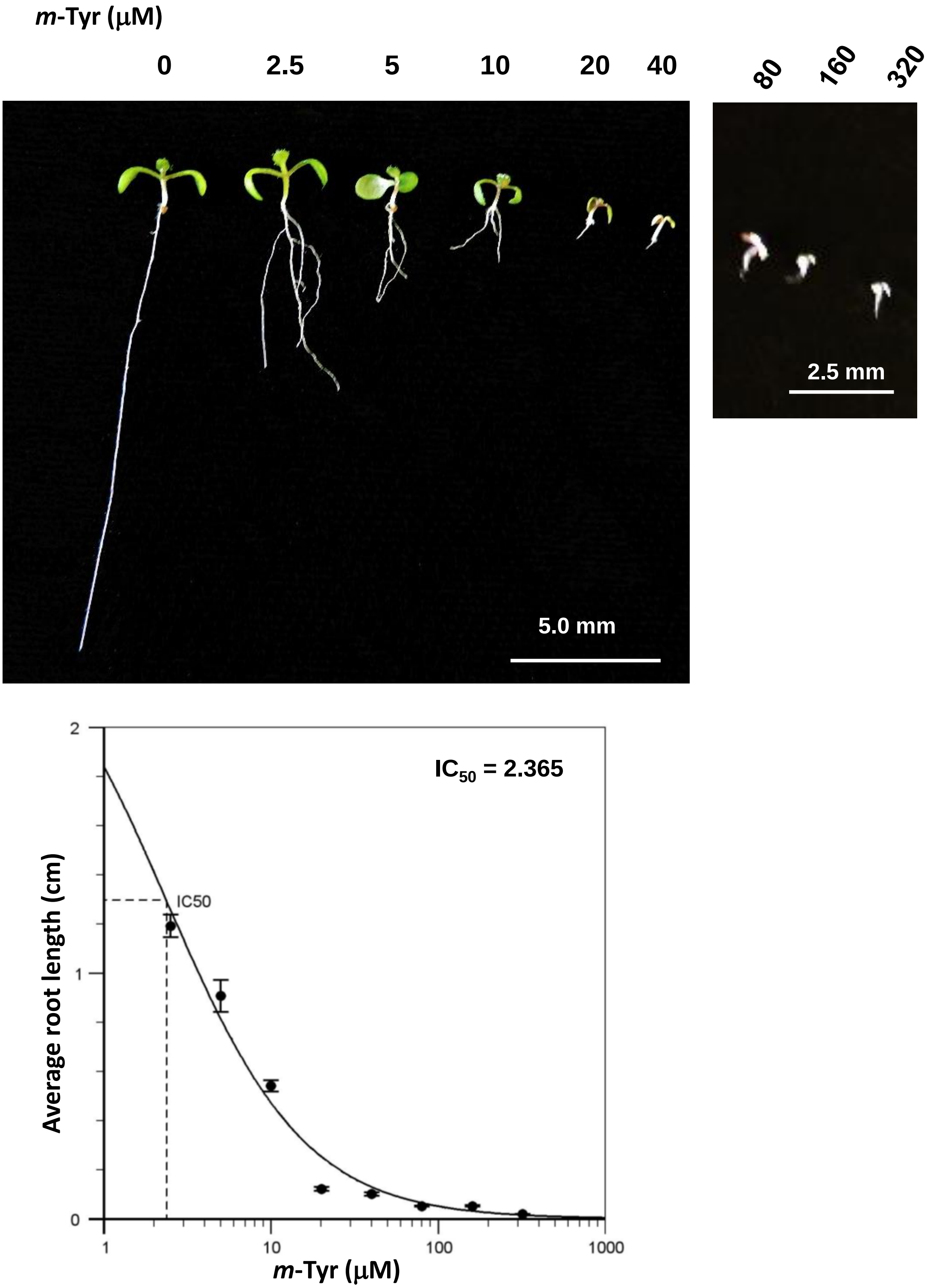

Supplemental Figure S1. Effects of *m*-tyrosine on early seedling development of *Arabidopsis thaliana* (Col-0) plants.

Arabidopsis thaliana seeds were sawn in MS-agar plates in absence or presence of different concentrations *m*-tyrosine (*m tyr*) as indicated. The figure shows 5 days old seedlings. IC_{50} (i.e., concentrations required to achieve 50% reduction of Arabidopsis root growth) was calculated from the relative root lengths of 5-day-old Arabidopsis seedlings grown at different m-tyrosine concentrations, using the 'Quest-Graph™ IC50 calculator' (https://www.aatbio.com/tools/ic50-calculator). The values are means of four biological replicates with about 25 seedlings in each treatment. Error bars indicate one standard deviation.
