# Supplementary material for: The Phytotoxicity of Meta-Tyrosine Is Associated With Altered Phenylalanine Metabolism and Misincorporation of This Non-Proteinogenic Phe-Analog to the Plant's Proteome
Source: Front Plant Sci. 2020 Mar 6;11:140. doi: 10.3389/fpls.2020.00140 (PMC7069529; doi:10.3389/fpls.2020.00140)
Supplement: Supplementary file 2 [file DataSheet_2.pdf]

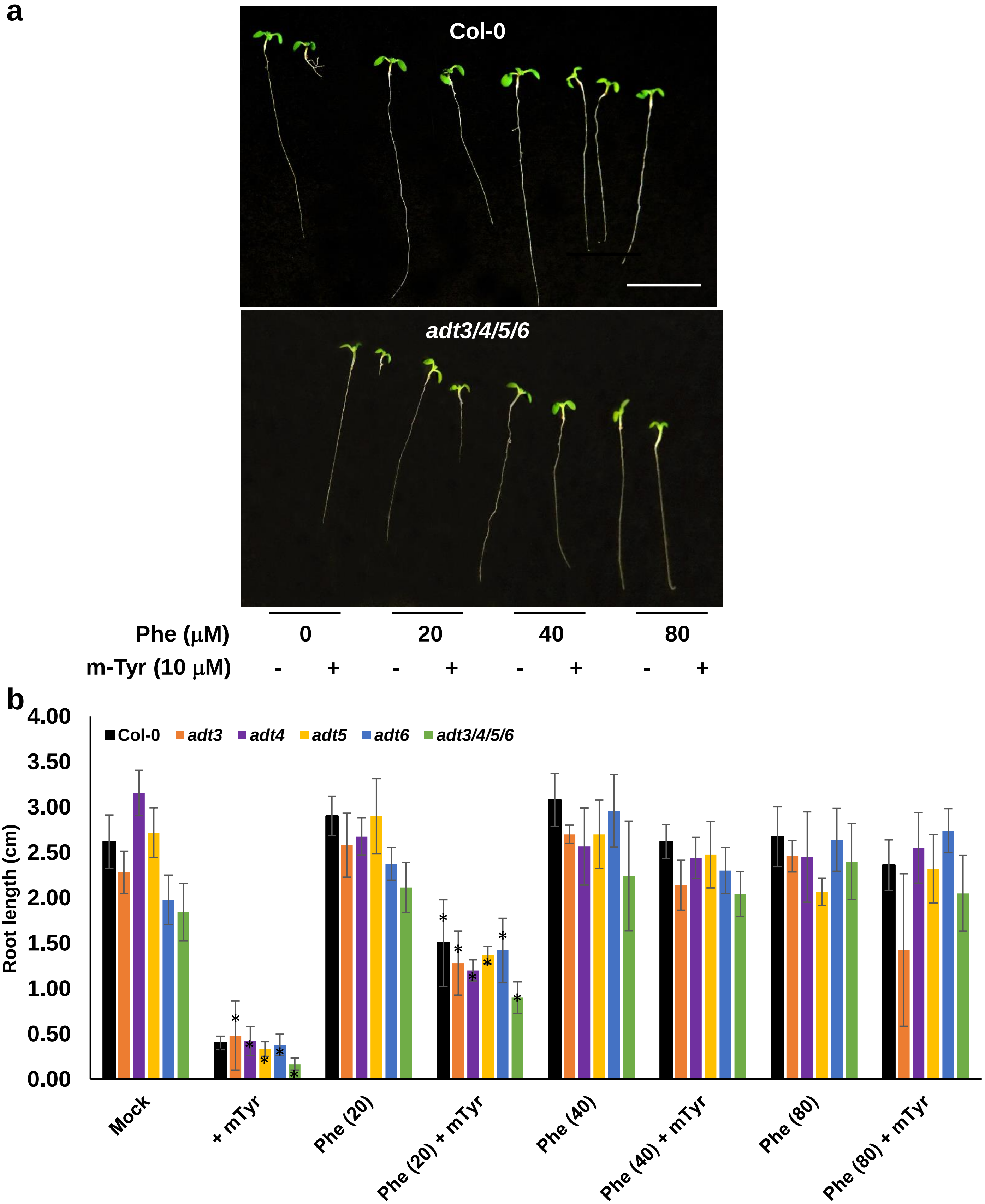

Supplemental Figure S2. The effect of phenylalanine on root growth of *Arabidopsis thaliana* wild-type (Col-0) and *adt3456* mutants grown in the absence or presence of *m*-tyrosine.

(A) 5-day-old Arabidopsis seeds (wild type and *adt3/4/5/6* mutant) were germinated in MS-agar plates in the absence (Mock) or presence of different Phe concentrations (20 -80 μM) and *m*-tyrosine (10 μM). (B) Measurements of the root lengths in 5-day-old Arabidopsis seedlings. Bar in panel A represents 1.0 cm. The values are means of three biological replicates with about 25 seedlings in each treatment. Error bars indicate one standard deviation. Asterisk in panel B indicates a significant difference from control (Student's T-test, P ≤ 0.05).
