# Supplementary material for: The Phytotoxicity of Meta-Tyrosine Is Associated With Altered Phenylalanine Metabolism and Misincorporation of This Non-Proteinogenic Phe-Analog to the Plant's Proteome
Source: Front Plant Sci. 2020 Mar 6;11:140. doi: 10.3389/fpls.2020.00140 (PMC7069529; doi:10.3389/fpls.2020.00140)
Supplement: Supplementary file 3 [file DataSheet_3.pdf]

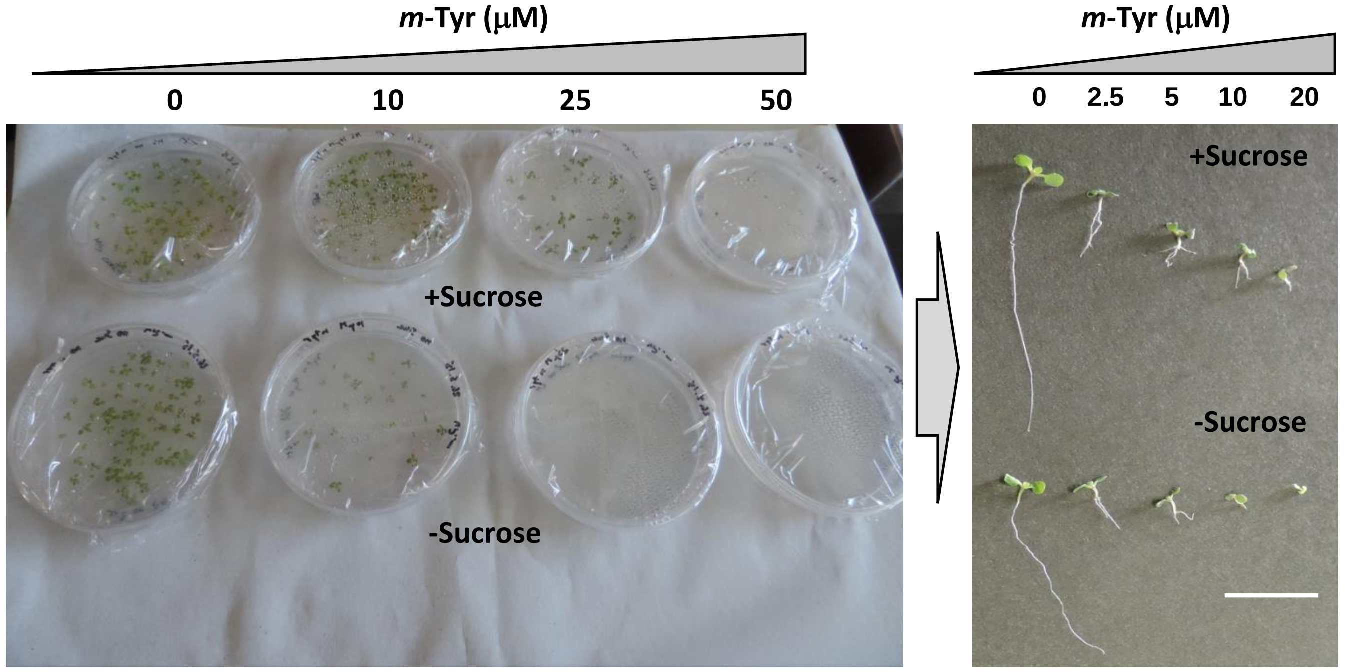

Supplemental Figure S3. The effects of m-tyrosine and sucrose on leaf coloring and seedling growth.
5-day-old Arabidopsis seedlings grown on MS plates containing different concentrations of *m*-tyrosine (*m*-Tyr) in the presence or absence of sucrose (1% w/v). Bar represents 1 cm.
