# Supplementary material for: The Phytotoxicity of Meta-Tyrosine Is Associated With Altered Phenylalanine Metabolism and Misincorporation of This Non-Proteinogenic Phe-Analog to the Plant's Proteome
Source: Front Plant Sci. 2020 Mar 6;11:140. doi: 10.3389/fpls.2020.00140 (PMC7069529; doi:10.3389/fpls.2020.00140)
Supplement: Supplementary file 4 [file DataSheet_4.pdf]

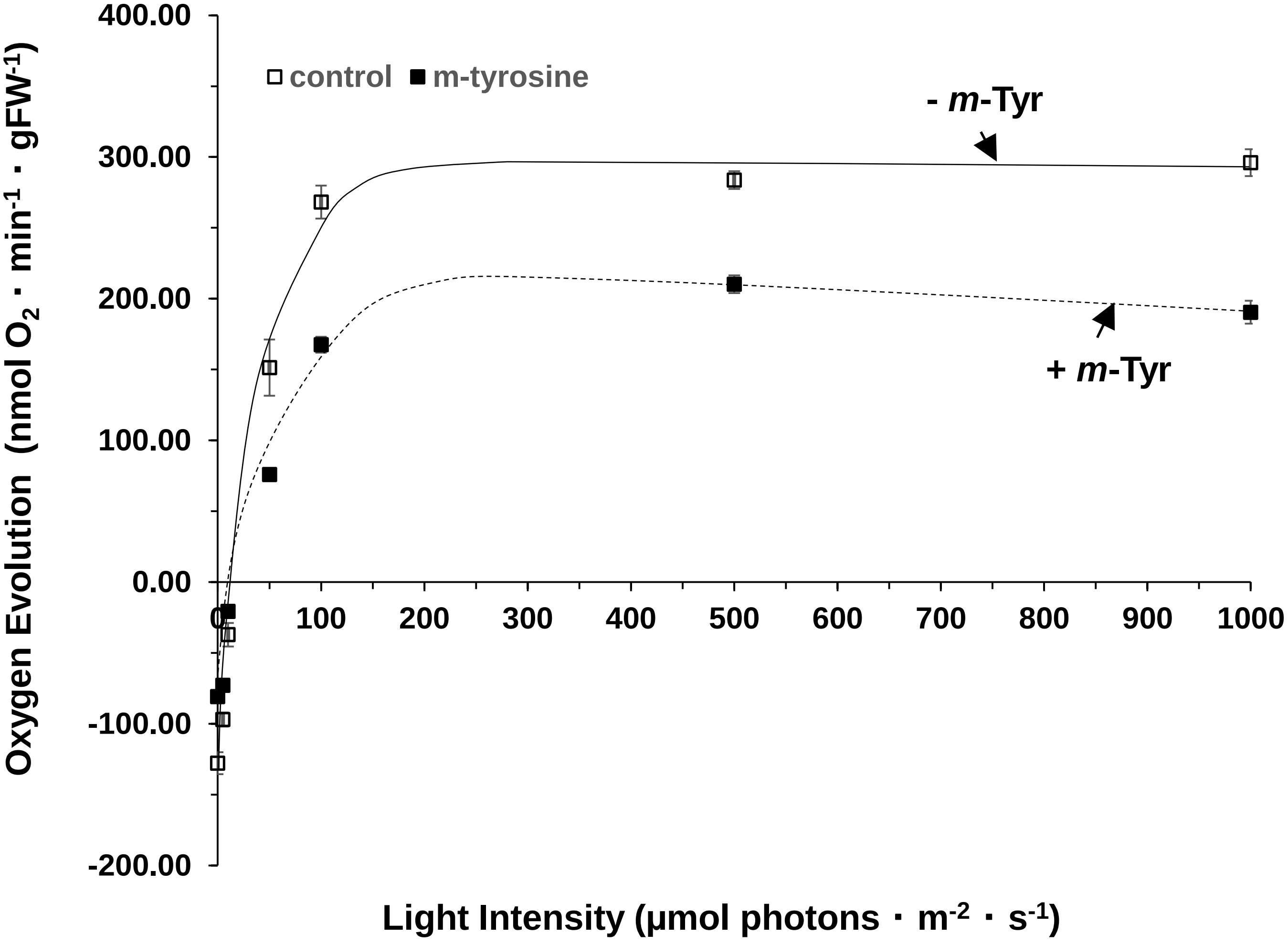

Supplemental Figure S4. The effect of *m*-tyrosine on respiration and photosynthetic activities of Arabidopsis plants.

Clark electrodes were used to measure the effect of intermittent light on photosynthetic oxygen evolution and dark respiration rates of 5-day-old Arabidopsis seedlings seeded on MS-agar plates in the absence (open boxes) or presence of 10 μM *m*-tyrosine (blacked boxes).
