# Supplementary material for: The Phytotoxicity of Meta-Tyrosine Is Associated With Altered Phenylalanine Metabolism and Misincorporation of This Non-Proteinogenic Phe-Analog to the Plant's Proteome
Source: Front Plant Sci. 2020 Mar 6;11:140. doi: 10.3389/fpls.2020.00140 (PMC7069529; doi:10.3389/fpls.2020.00140)
Supplement: Supplementary file 5 [file DataSheet_5.pdf]

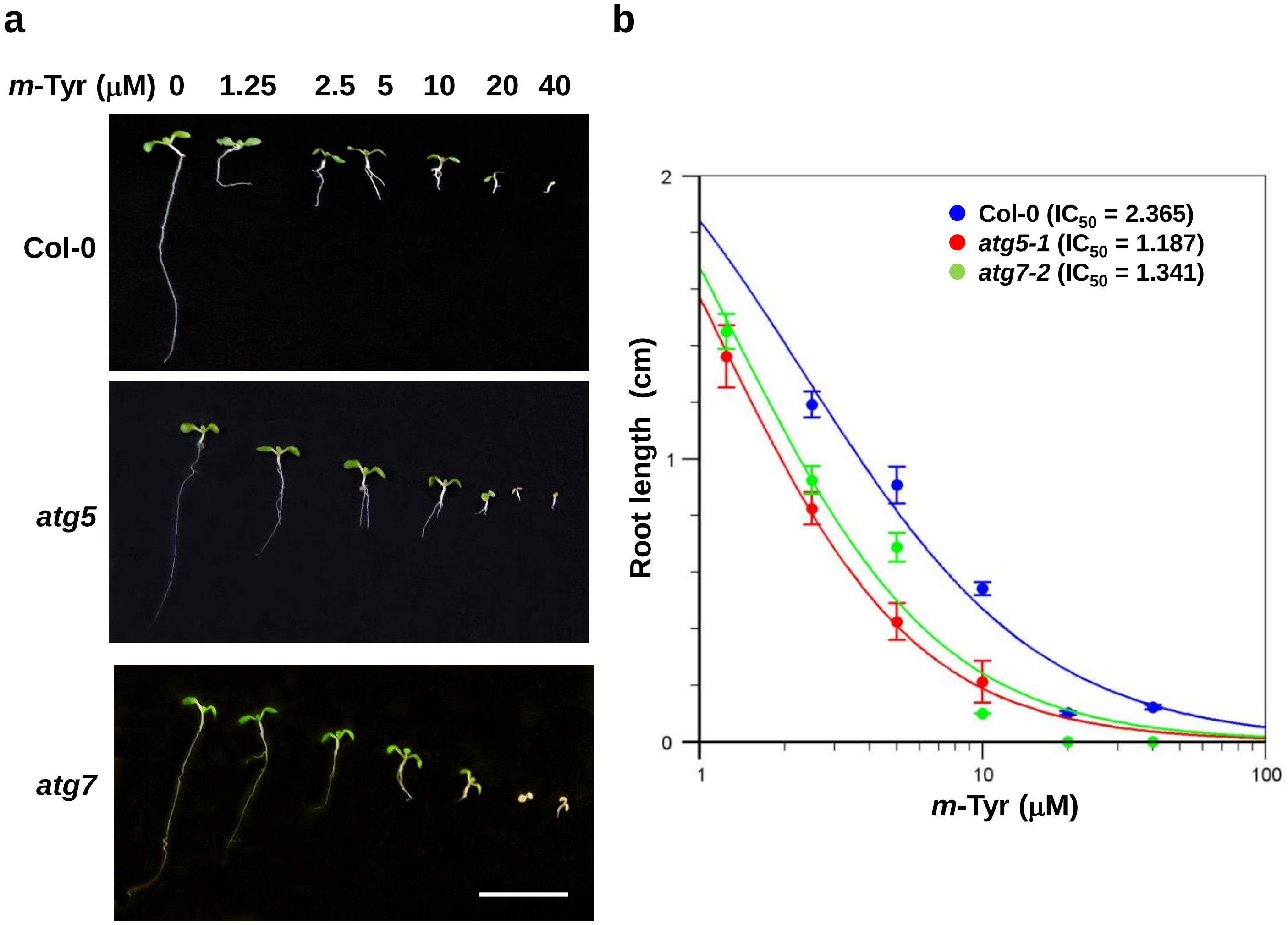

Supplemental Figure S5. The effect of *m*-tyrosine on root growth of *Arabidopsis thaliana* wild-type and *atg* mutants.

(A) 5-day-old Arabidopsis seeds of wild-type (Col-0), *atg5 and atg7* mutant-lines, were germinated in MS-agar plates in the absence ('0') or presence of *m*-tyrosine (1.25 to 40 μM). Bar in panel A represents 1.0 cm. (B) Measurements of the root lengths in 5-day-old Arabidopsis seedlings. IC_{50} (i.e., concentrations required to achieve 50% reduction of Arabidopsis root growth) was calculated from the relative root lengths of 5-day-old Arabidopsis seedlings grown at different *m*-tyrosine concentrations, using the 'Quest-Graph™ IC50 calculator' (https://www.aatbio.com/tools/ic50-calculator). The values are means of four biological replicates with about 25 seedlings in each treatment. Error bars indicate one standard deviation. Asterisk in panel B indicates a significant difference from control (Student's T-test, P ≤ 0.05).
