# Supplementary material for: The Phytotoxicity of Meta-Tyrosine Is Associated With Altered Phenylalanine Metabolism and Misincorporation of This Non-Proteinogenic Phe-Analog to the Plant's Proteome
Source: Front Plant Sci. 2020 Mar 6;11:140. doi: 10.3389/fpls.2020.00140 (PMC7069529; doi:10.3389/fpls.2020.00140)
Supplement: Supplementary file 6 [file Table_1.pdf]

Supplemental Table S1. Antibodies used for the analysis of *m*-tyrosine effects on Arabidopsis.

**Antibody**	**Protein I.D.**	**origin**	**serum**	**dilution**	**Reference / source**
AOX1/2	Alternative oxidase subunits1/2	*Typhonium venosum*	Mouse (monoclonal)	1 : 5,00	(Elthon *et al.* 1989)
AtpA	Mitochondrial ATP-synthase subunit α	*Zea mays*	Mouse (monoclonal)	1 : 5,000	(Michael *et al.* 1993)
CA2	γ-carbonic anhydrase-like subunit 2	*Arabidopsis thaliana*	Rabbit (polyclonal)	1 : 1,000	(Perales *et al.* 2005, Sunderhaus *et al.* 2006)
COX2	Cytochrome oxidase subunit-2	*Arabidopsis thaliana*	Rabbit (polyclonal)	1 : 5,000	Agrisera antibodies, AS04 053A
NAD9	NADH-dehydrogenase complex subunit-9	*Triticum spp.*	Rabbit (polyclonal)	1 : 50,000	(Lamattina *et al.* 1993)
NDB2	external NADH-dehydrogenase subunit 2	*Arabidopsis thaliana*	Rabbit (polyclonal)	1 : 2,000	(Carrie *et al.* 2008)
RISP	Rieske iron-sulfur protein	*Arabidopsis thaliana*	Rabbit (polyclonal)	1 : 5,000	Gift of Prof. Ian Small, UWA
VDAC	Voltage-dependent anion channel	*Zea mays*	Mouse (monoclonal)	1 : 5,000	Thomas Elthon collection, PM035
PsaA	Photosystem I P700 chlorophyll a apoprotein A1	*Chlamydomonas reinhardtii*	Rabbit (polyclonal)	1 : 1,000	Agrisera (AS06 172)
PsbA	Photosystem II D1 protein	synthetic peptide	Rabbit (polyclonal)	1 : 500	Agrisera (AS05 084)
PsbC	Photosystem II CP43 reaction center protein	*Arabidopsis thaliana*	Rabbit (polyclonal)	1 : 1,000	Gift of Prof. Nir Keren (HUJI)
PsbD	Photosystem II D2 protein	*Arabidopsis thaliana*	Rabbit (polyclonal)	1 : 1,000	Gift of Prof. Nir Keren (HUJI)

References

Carrie, C., Murcha, M.W., Kuehn, K., Duncan, O., Barthet, M., Smith, P.M., Eubel, H., Meyer, E., Day, D.A., Millar, A.H. and Whelan, J. (2008) Type II NAD(P)H dehydrogenases are targeted to mitochondria and chloroplasts or peroxisomes in Arabidopsis thaliana. *FEBS Lett*, **582**, 3073-3079.

Elthon, T.E., Nickels, R.L. and McIntosh, L. (1989) Monoclonal antibodies to the alternative oxidase of higher plant mitochondria. *Plant Physiol*, **89**, 1311-1317.

Lamattina, L., Gonzalez, D., Gualberto, J. and Grienenberger, J.-M. (1993) Higher plant mitochondria encode an homologue of the nuclear-encoded 30-kDa subunit of bovine mitochondrial complex I. *Eur J Biochem*, **217**, 831-838.

Michael, H.L., Arnost, H. and Elthon, T.E. (1993) Monoclonal Antibodies to the a- and b-Subunits of the Plant Mitochondrial F_1-ATPase. *Plant Physiology*, **101**, 931-937.

Perales, M., Eubel, H., Heinemeyer, J., Colaneri, A., Zabaleta, E. and Braun, H.-P. (2005) Disruption of a nuclear gene encoding a mitochondrial gamma carbonic anhydrase reduces complex I and supercomplex I+III2 levels and alters mitochondrial physiology in Arabidopsis. *J Mol Biol*, **350**, 263-277.

Sunderhaus, S., Dudkina, N.V., Jansch, L., Klodmann, J., Heinemeyer, J., Perales, M., Zabaleta, E., Boekema, E.J. and Braun, H.P. (2006) Carbonic anhydrase subunits form a matrix-exposed domain attached to the membrane arm of mitochondrial complex I in plants. *J Biol Chem*, **281**, 6482-6488.
