# Supplementary material for: The Phytotoxicity of Meta-Tyrosine Is Associated With Altered Phenylalanine Metabolism and Misincorporation of This Non-Proteinogenic Phe-Analog to the Plant's Proteome
Source: Front Plant Sci. 2020 Mar 6;11:140. doi: 10.3389/fpls.2020.00140 (PMC7069529; doi:10.3389/fpls.2020.00140)
Supplement: Supplementary file 7 [file Table_2.pdf]

**Supplemental Table S2.** Profiling of native amino acids in 5-day-old *Arabidopsis thaliana* seedlings grown in the absence (untreated) or presence of 10 μM *m*-tyrosine (*m*-Tyr).

| | **Amino acid** | **control** | **+ *m*-Tyr** |
|---|---|---|---|
| 1 | Alanine | 1.00 ± 0.12 | 0.82 ± 0.13 |
| 2 | Arginine | 1.00 ± 0.15 | 0.78 ± 0.16 |
| 3 | Asparagine | 1.00 ± 0.17 | 0.79 ± 0.18 |
| 4 | Aspartate | 1.00 ± 0.09 | 0.74 ± 0.16 |
| 5 | Cysteine | 1.00 ± 0.14 | 0.92 ± 0.24 |
| 6 | Glutamate | 1.00 ± 0.13 | 1.01 ± 0.14 |
| 7 | Glutamine | 1.00 ± 0.16 | 1.03 ± 0.29 |
| 8 | Glycine | 1.00 ± 0.07 | 0.78 ± 0.08 |
| 9 | Histidine | 1.00 ± 0.19 | 0.79 ± 0.21 |
| 10 | Isoleucine | 1.00 ± 0.06 | 0.85 ± 0.16 |
| 11 | Leucine | 1.00 ± 0.07 | 0.79 ± 0.22 |
| 12 | Lysine | 1.00 ± 0.11 | 0.79 ± 0.23 |
| 13 | Methionine | 1.00 ± 0.10 | 0.75 ± 0.16 |
| 14 | Phenylalanine | 1.00 ± 0.11 | **0.57 ± 0.06** |
| 15 | Proline | 1.00 ± 0.13 | 1.11 ± 0.18 |
| 16 | Serine | 1.00 ± 0.17 | 0.77 ± 0.21 |
| 17 | Threonine | 1.00 ± 0.07 | 0.79 ± 0.21 |
| 18 | Tryptophan | 1.00 ± 0.09 | 0.86 ± 0.23 |
| 19 | Tyrosine | 1.00 ± 0.22 | 0.64 ± 0.12 |
| 20 | Valine | 1.00 ± 0.10 | 0.94 ± 0.15 |

5-day-old seedlings grown in the absence or presence of 10 μM exogenous *m*-tyrosine (*m*-Tyr) were analyzed by GC-qTOF-MS. Data is presented as averages of 4 replicates normalized to control plants (i.e., untreated plants germinated in MS-media), grown under the same growth conditions. The data shows the relative pick area of each amino acid in untreated versus *m*-tyrosine-treated seedlings. The values are means of four biological replicates ± SE. Significant differences following student's t-test ($P \leq 0.05$), compared to the control, are represented in bold.
