# Supplementary material for: The Phytotoxicity of Meta-Tyrosine Is Associated With Altered Phenylalanine Metabolism and Misincorporation of This Non-Proteinogenic Phe-Analog to the Plant's Proteome
Source: Front Plant Sci. 2020 Mar 6;11:140. doi: 10.3389/fpls.2020.00140 (PMC7069529; doi:10.3389/fpls.2020.00140)
Supplement: Supplementary file 8 [file Table_3.pdf]

Experiment title: Free Amino Acids measurements of m-Tyr treated Arabidopsis seedlings
Organism/Plant species: Arabidopsis thaliana seedlings
Organ/tissue: 5-day-old whole seedlings
Analytical tool: GC-qTOF-MS

Peak/compound #- number of compound found
RT - retention time
Putative Name- putative identification of the metabolite/derivative
Corresponding metabolite name in literature
Mol. Formula- molecular formula of the metabolite
Mass to charge ratio (m/z)
Standard - Standard compound used for idnetification (Sigma catalog number)

Peak/compound #	Putative name	Metabolite name	Moleclar formula	m/z	RT(min)	Metabolite class
1	L-Alanine, 2TMS	Alanine	$C_3H_7NO_2$	116.0892	4.667	Amino acid
2	Alanine (3TMS)	Alanine	$C_3H_7NO_2$	188.126	7.233	Amino acid
3	Arginine, 3TMS	Arginine	$C_6H_{14}N_4O_2$	157.124	11.302	Amino acid
4	Asparagine (4TMS)	Asparagine	$C_4H_8N_2O_3$	216.1254	9.054	Amino acid
5	Asparagine, 3TMS	Asparagine	$C_4H_8N_2O_3$	116.0915	10.458	Amino acid
6	L-Aspartic acid, 2TMS	Aspartic Acid	$C_4H_7NO_4$	160.081	8.568	Amino acid
7	L-Aspartic acid, 3TMS	Aspartic Acid	$C_4H_7NO_4$	232.1168	8.907	Amino acid
8	Cysteine, 3TMS	Cysteine	$C_3H_7NO_2S$	100.065	6.985	Amino acid
9	L-Glutamic acid, 3TMS	Glutamic acid	$C_5H_9NO_5$	246.1336	9.738	Amino acid
10	L-Glutamine, 3TMS	Glutamine	$C_5H_{10}N_2O_3$	156.0778	11.256	Amino acid
11	Glycine, 3TMS	Glycine	$C_2H_5NO_2$	174.1269	6.735	Amino acid
12	L-Histidine, 3TMS	Histidine	$C_6H_9N_3O_2$	154.525	14.723	Amino acid
13	L-isoleucine TMS	Isoleucine	$C_6H_{13}NO_2$	188.1089	6.058	Amino acid
14	L-Isoleucine, 2TMS s	Isoleucine	$C_6H_{13}NO_2$	158.1399	6.615	Amino acid
15	L-Leucine, TMS	Leucine	$C_6H_{13}NO_2$	86.0959	5.772	Amino acid
16	Lysine (4TMS)	Lysine	$C_6H_{14}N_2O_2$	317.2294	11.551	Amino acid
17	L-Methionine, 2TMS	Methionine	$C_5H_{11}NO_2S$	176.0909	9.241	Amino acid
18	Phenylalanine, 2TMS	Plenylalanine	$C_9H_{11}NO_2$	218.1079	10.197	Amino acid
19	Proline (1TMS)	Porline	$C_5H_9NO_2$	70.0671	6.309	Amino acid
20	L-Proline, 2TMS	Proline	$C_5H_9NO_2$	142.1065	6.949	Amino acid
21	L-Serine	Serine	$C_3H_7NO_3$	116.052	6.751	Amino acid
22	Serine, 3TMS	Serine	$C_3H_7NO_3$	204.1271	7.236	Amino acid
23	L-Threonine, 2TMS	Threonine	$C_4H_9NO_3$	130.069	7.015	Amino acid
24	L-Threonine, 3TMS	Threonine	$C_4H_9NO_3$	218.1351	7.405	Amino acid
25	L-Tryptophan, 3TMS	Tryptophane	$C_{11}H_{12}N_2O_2$	202.1016	14.523	Amino acid
26	L-Tyrosine, 3TMS	Tyrosine	$C_9H_{11}NO_3$	280.1575	12.283	Amino acid
27	L-Valine, TMS	Valine	$C_5H_{11}NO_2$	72.0825	5.059	Amino acid
28	L-Valine, 2TMS	Valine	$C_5H_{11}NO_2$	144.1201	5.814	Amino acid
29	Ribitol	Ribitol	$C_5H_{12}O_5$	319.1626	9.606	Sugar alcohol
